# Dopamine D3 receptor blockade restores hippocampal synaptic plasticity and rescues memory deficits in Alzheimer's disease mouse models

**DOI:** 10.3389/fnagi.2026.1840697

**Published:** 2026-07-13

**Authors:** Maria Rosaria Tropea, Giuseppe Aceto, Roberta Carmela Trovato, Emanuele Nicitra, Nicole Santuccio, Giorgia Intili, Marcello D'Ascenzo, Daniela Puzzo

**Affiliations:** 1 Department of Biomedical and Biotechnological Sciences, University of Catania, Catania, Italy; 2 Oasi Research Institute-IRCCS, Troina, Italy; 3 Department of Neuroscience, Università Cattolica del Sacro Cuore, Rome, Italy; 4 Fondazione Policlinico Universitario A. Gemelli IRCCS, Rome, Italy

**Keywords:** Alzheimer's disease, cariprazine, dopamine D3 receptors, hippocampus, memory, synaptic plasticity

## Abstract

**Introduction:**

Early synaptic failure is widely considered a primary driver of cognitive decline in Alzheimer's disease (AD), and previous studies have suggested that dopaminergic signaling may contribute to hippocampal synaptic dysfunction. Among dopaminergic receptors, dopamine D3 receptors (D3Rs) have emerged as important modulators of synaptic plasticity and cognitive processes, but their role in AD-related synaptic impairment remains unclear. The aim of this study was to determine whether pharmacological blockade of D3Rs could restore memory deficits and hippocampal synaptic dysfunction in preclinical models of AD.

**Methods:**

Behavioral studies, including novel object recognition (NOR), novel object location (NOL), and open-field tests, were performed to evaluate recognition memory, spatial memory, locomotor activity, and anxiety-related behavior in two mechanistically distinct mouse models of AD: triple-transgenic 3xTg-AD mice and α7 nicotinic acetylcholine receptor knockout (α7KO) mice. Electrophysiological recordings in hippocampal slices were used to assess AMPA/NMDA ratio, basal synaptic transmission, and long-term potentiation (LTP). qPCR and western blot analyses were performed to evaluate hippocampal D3R mRNA and protein expression, respectively. Pharmacological treatments included the selective D3R antagonist NGB-2904 and cariprazine, a clinically approved antipsychotic with high affinity for D3Rs.

**Results:**

Recognition and spatial memory deficits were rescued by NGB-2904 and cariprazine in both AD models, without affecting locomotor activity or anxiety-related behavior. No sex-dependent differences were observed in the behavioral response to treatment. Electrophysiological recordings revealed a reduced AMPA/NMDA ratio and impaired LTP in both models, while basal synaptic transmission was selectively reduced in 3xTg-AD mice. D3R-targeting compounds restored synaptic transmission and plasticity. Inhibition of PKA prevented the rescue of LTP induced by D3R blockade, suggesting the involvement of the cAMP/PKA signaling pathway. Both models displayed reduced hippocampal D3R mRNA expression and protein levels, suggesting that the residual population of D3Rs might represent a viable target.

**Conclusion:**

Together, these findings demonstrate that D3R-targeting compounds rescue synaptic plasticity and memory deficits in two mechanistically distinct AD models. These results support a role for dopaminergic signaling in early synaptic dysfunction and highlights D3R modulation as a relevant pathway for further investigation in AD-related cognitive impairment.

## 1 Introduction

Alzheimer's disease (AD) is a chronic and progressive neurodegenerative disorder and the leading cause of dementia in the elderly population worldwide. It is clinically characterized by a gradual and irreversible decline in memory and other cognitive functions, ultimately resulting in severe functional impairment and loss of independence. Despite decades of intense research, there are currently no disease-modifying therapies capable of preventing, halting, or reversing disease progression.

Although amyloid-β plaques and tau neurofibrillary tangles represent the major histopathological hallmarks of AD, it is now widely recognized that synaptic dysfunction precedes neurodegeneration and is more closely associated with cognitive decline (Gulisano et al., 2018). In this context, growing attention has been directed toward the role of neuromodulatory systems, which critically regulate synaptic plasticity, learning, and memory.

The dopaminergic system has emerged as an important contributor to AD pathophysiology (Moreira et al., 2025; Zhang et al., 2025). Preclinical studies have shown that pharmacological or genetic manipulations that increase dopaminergic tone improve memory performance in several animal models of AD (Ambrée et al., 2009; Guzmán-Ramos et al., 2012; Jürgensen et al., 2011; La Barbera et al., 2021; Yuan Xiang et al., 2016). Consistently, a selective loss of dopaminergic neurons in the ventral tegmental area (VTA) has been reported at early stages of the disease in AD mouse models (Nobili et al., 2017). In humans, alterations of the dopaminergic system have also been associated with AD and with deficits in motivation, executive function, and memory (Pan et al., 2019). Neuroimaging studies have revealed structural and functional alterations of the VTA and its connectivity with other brain regions in AD (De Marco and Venneri, 2018; Sala et al., 2021; Serra et al., 2021), and genetic studies have identified polymorphisms affecting dopaminergic transmission that result in reduced dopamine levels and increased risk of AD (Roussotte et al., 2015).

While most studies have focused on D1-class dopamine receptors, accumulating evidence indicates that D3 dopamine receptors (D3Rs), metabotropic receptors of the D2-like family, play a critical role in the regulation of synaptic plasticity and cognitive processes.

D3Rs are highly expressed in mesocortical and limbic regions, including the hippocampus, where they modulate glutamatergic excitatory neurotransmission (Sokoloff et al., 2013), a process critical for memory formation. At the synaptic level, D3Rs act presynaptically as autoreceptors to regulate dopamine release (Stahl, 2017), and are also abundantly expressed on glutamatergic postsynaptic dendrites, supporting their involvement in synaptic plasticity and memory (Tropea et al., 2024). Studies in both animal models and humans have demonstrated that D3R blockade enhances cognitive performance (Glickstein et al., 2005; Gross et al., 2013; Laszy et al., 2005; Loiseau and Millan, 2009; Tropea et al., 2024; Xing et al., 2010), whereas receptor activation impairs multiple cognitive domains (Nakajima et al., 2013; Ukai et al., 1997; Watson et al., 2012). Consistently, dysregulated D3R signaling has been reported in several neuropsychiatric and neurodegenerative conditions (Torrisi et al., 2023), yet its specific contribution to AD-related cognitive dysfunction remains largely unexplored.

Recently, it was shown that genetic deletion or pharmacological blockade of D3Rs rescues the impairment of synaptic transmission, long-term potentiation (LTP), and memory in aged mice, supporting the potential relevance of D3R targeting to counteract age-related hippocampal cognitive decline (Tropea et al., 2024). Whether a similar mechanism may counteract AD-related synaptic dysfunction, however, remains unknown. Based on these findings, the aim of the present study was to determine whether pharmacological blockade of D3Rs could restore hippocampal memory and synaptic plasticity deficits in two mechanistically distinct mouse models of AD to investigate the contribution of D3R signaling across different pathogenic contexts.

Specifically, we used the 3xTg-AD mouse model that harbors three human mutations associated with familial AD (APP_Swe_, PSEN1_M146V_, and MAPT_P301L_) and recapitulates key histopathological and cognitive features of the disease, including amyloid and tau pathology (Oddo et al., 2003). Although familial AD represents only a small fraction of total cases, this model remains a well-established model for mechanistic and preclinical studies.

To complement this genetic model, we also used α7 nicotinic acetylcholine receptor knockout (α7KO) mice, which model cholinergic dysfunction independently of genetically driven amyloid overproduction. α7nAChRs occupy a strategic position within hippocampal circuits because they regulate both glutamatergic and dopaminergic neurotransmission. Through their high calcium permeability and pre- and postsynaptic localization, α7nAChRs facilitate glutamate release, modulate AMPAR- and NMDAR-dependent signaling, and promote dopamine release in cortical and limbic regions (Besson et al., 2012; Cheng and Yakel, 2015; Quarta et al., 2007). In parallel, D2-like dopamine receptors, including D2Rs and D3Rs, modulate glutamatergic transmission and synaptic plasticity through both presynaptic and postsynaptic mechanisms (Sokoloff et al., 2013; Tropea et al., 2024). These reciprocal interactions establish a bidirectional crosstalk between cholinergic, dopaminergic, and glutamatergic signaling pathways that is essential for hippocampal plasticity and memory formation. Disruption of this integrated network may therefore contribute to the early synaptic dysfunction observed in AD. In a previous study, we demonstrated that genetic deletion of α7nAChRs leads to the progressive development of synaptic, cognitive, and molecular alterations resembling AD, resulting in an age-dependent AD-like phenotype (Tropea et al., 2021). Therefore, the α7KO model provides an opportunity to determine whether the beneficial effects of D3R antagonism extend beyond amyloid-driven pathology and are also observed in the context of cholinergic impairment and sporadic-like pathogenic mechanisms.

## 2 Materials and methods

### 2.1 Animals

We used wild type (WT) mice (C57BL/6J; RRID:IMSR_JAX:000664), 3xTg-AD mice [(B6;129-Tg(APPSwe, tauP301L)1Lfa; RRID:MMRRC_034830-JAX], and α7KO mice (B6.129S7-Chrna7tm1Bay/J; RRID:IMSR_JAX:003232) purchased from The Jackson Laboratory. Colonies were established in the animal facilities at University of Catania, and Università Cattolica del Sacro Cuore. The housing conditions were controlled, maintaining stable hygrometric and thermic conditions (50 %; 21 °C ± 1 °C) on a 12-h light/dark cycle with ad *libitum* access to food and water.

Animal care, handling, and procedures were carried out in accordance with national (D.L. n. 26, 14 March 2014) and European Community Council Directives (2010/63/UE) and were approved by the Italian Ministry of Health (226/2021-PR, 944/2021-PR). The experiments complied with the ARRIVE guidelines and were conducted to minimize animal suffering.

A total of 229 animals (*n* = 73 WT, *n* = 78 3xTg-AD, and *n* = 78 α7KO mice) were used in this study. Animals were randomly assigned to treatment groups and experimental procedures balancing genotype, age and sex where appropriate, in order to reduce potential bias and ensure consistency across experiments. Sample size was determined based on previous studies using the same animal models and experimental paradigms, considering the expected effect size, experimental variability, and the principle of minimizing animal use.

Male mice were used for electrophysiological recordings, whereas sex-balanced cohorts were used for behavioral experiments, qPCR analyses, and Western blot assays. The number of male and female animals included in each experimental group is reported in Supplementary Figure 1.

Animals were studied at ages corresponding to the onset of AD-related phenotypic alterations in each model: 10–12 months for 3xTg-AD mice, and 12–14 months for α7KO mice, based on previous reports describing age-dependent pathological and cognitive changes in these models (Oddo et al., 2003; Tropea et al., 2021). WT mice at 10–14 months were used as controls.

A schematic overview of the experimental design and timeline of experiments is provided in Supplementary Figure 1.

### 2.2 Drugs

As D3R-targeting compounds, we used the selective D3 receptor antagonist NGB-2904 (Ki = 1.4 nM for D3Rs) and cariprazine, a clinically approved D2/D3 partial agonist with preferential affinity for D3Rs (Ki = 0.09 nM for D3Rs).

For behavioral experiments, NGB-2904 (Sigma-Aldrich) and cariprazine (MedChemExpress) were first dissolved in a vehicle containing DMSO and Tween-20 and then diluted in saline solution (0.9% NaCl). The final DMSO concentration never exceeded 0.1%. Final doses were 3 mg/kg for NGB-2904 (Tropea et al., 2024) and 0.01 mg/kg for cariprazine (Caccia et al., 2013), administered intraperitoneally (i.p.) in a final volume of 200 μl. NGB-2904 was administered 20 min prior to the training phase (T1) of novel object recognition (NOR) and novel object location (NOL) or the open field test, whereas cariprazine was administered 30 min prior to T1 or open field test.

For electrophysiological recordings, hippocampal slices were treated with NGB-2904 (1 μM) or cariprazine (1 nM). Stock solutions were prepared in DMSO and aliquots were diluted in artificial cerebrospinal fluid (ACSF) to the desired final concentration immediately before recordings. Slices were perfused with NGB-2904 for 15 min prior to AMPA/NMDA recordings, basal synaptic transmission assessment, or LTP induction. Cariprazine was applied 20 min before basal synaptic transmission assessment or LTP induction. For experiments assessing the involvement of the cAMP/PKA pathway, slices were perfused with Rp-8-Br-cAMPS (Biolog, 20 μM) for 30 min before tetanus and for 15 min thereafter.

### 2.3 Behavioral studies

#### 2.3.1 Novel object recognition and location

NOR and NOL tests were performed as previously described (Tropea et al., 2021). We used an apparatus of our design and construction, consisting of an arena (45 × 45 × 40 cm) made by white matte polymethyl methacrylate non-reflective panels illuminated by a perpendicular light source located 65 cm from the floor (Tropea et al., 2022). A webcam, connected to the computer, was fixed on the top of the apparatus. After 5 days of habituation to the arena, objects, and i.p. injections, mice underwent the training session (T1), and after 24 h, the testing session (T2) was conducted to assess memory retention. During T1, mice were placed in the arena and allowed to explore two identical objects for 10 min. In T2 (10 min), for NOR, mice were presented with two different objects, a “familiar” one (i.e., the one used in T1) and a “novel” one; for NOL, mice were presented with an object located as in T1, and the other in a different location. For both tests, animal exploration—defined as the mouse pointing its nose toward the object from a distance not > 2 cm—was measured in T2. We measured: (i) the percentage of time spent exploring each of the two identical (or in the same position) objects during T1; (ii) total exploration time during T1 and T2; and (iii) the percentage of time spent exploring the familiar and novel object/location during T2. The discrimination (D) index, “exploration of novel object minus exploration of familiar object/total exploration time” was analyzed. Mice with a total exploration time <5 s were excluded from analysis.

#### 2.3.2 Open field

Open field test was conducted in the same apparatus used for the NOR and NOL tasks, but under increased illumination to promote anxiety-like behavior (Tropea et al., 2022). Each mouse was placed in the arena and allowed to freely explore for 5 min. We scored the following parameters: i) % time spent in the center ii) number of entries into the center with four paws.; iii) total locomotor activity (calculated as the time spent moving into the arena plus the time spent erected on its hind legs); iv) freezing (time of immobility) as previously described (Palmeri et al., 2016).

### 2.4 Elecrophysiological recordings

#### 2.4.1 Patch-clamp recordings

For brain slice preparation, we followed the protocol described in Aceto et al., 2022, with minor modifications. After cervical dislocation, brains were rapidly removed and placed in ice-cold, sucrose-based cutting solution containing the following (in mM): TRIS-HCl 72, TRIZMA base 18, NaH_2_PO_4_ 1.2, NaHCO_3_ 30, KCl 2.5, glucose 25, HEPES 20, MgSO_4_ 10, Na-pyruvate 3, ascorbic acid 5, CaCl_2_ 0.5, sucrose 20. Slices (300 μm thick) were cut on a vibratome (VT1200S; Leica Microsystems, Germany) and immediately transferred to an incubation chamber held at 32 °C and filled with a recovery solution containing (in mM): TRIS-HCl 72, TRIZMA base 18, NaH_2_PO_4_ 1.2, NaHCO_3_ 25, KCl 2.5, glucose 25, HEPES 20, MgSO_4_ 10, Na-pyruvate 3, ascorbic acid 5, CaCl_2_ 0.5, sucrose 20. After 30 min, slices were transferred to a second incubation chamber held at 32 °C containing artificial cerebrospinal fluid (aCSF) with (in mM): NaCl 124, KCl 3.2, NaH_2_PO_4_ 1.2, MgCl_2_ 1, CaCl_2_ 2, NaHCO_3_ 26 and glucose 10, pH 7.4. During incubations, the chambers were continuously bubbled with 95% O_2_/5% CO_2_. Finally, slices were equilibrated at RT for at least 30 min. Slices were then transferred to a submerged recording chamber constantly perfused with heated aCSF (30–32 °C) and bubbled with 95% O_2_/5% CO_2_. Neurons of the CA1 area were visualized under DIC infrared illumination. Stimulation of the SC was obtained by means of a current stimulus isolator (WPI, Worcester, MA, USA), connected to a bipolar concentric stimulating electrode (FHC, Bowdoin, ME, USA) which was positioned in contact with the SC pathway. Patch pipettes had a resistance of 4–6 MΩ when filled with an internal solution containing (in mM): CsCH_3_SO_3_ 135, HEPES 10, NaCl 8, EGTA 0.25, MgCl_2_ 2, Mg-ATP 4, Na-GTP 0.3, phosphocreatine 5, pH adjusted to 7.3 with NaOH. After establishing a gigaseal, whole-cell configuration was achieved by applying negative pressure. A series resistance lower than 15 MΩ was considered acceptable and monitored throughout the recording, and experiments were discarded if changes exceeded 20%. For evoked and spontaneous excitatory postsynaptic currents (EPSC) measurements, neurons were held at −70 mV and electrical stimuli were delivered to the SC. To obtain the AMPA/NMDA currents ratio, stimuli of identical amplitude were delivered at holding potentials of −70 and +40 mV, as described (Ahmad et al., 2012), with a frequency of 0.05 Hz; 50 μM picrotoxin (PTX) was added to the bath. The identity of evoked EPSCs was confirmed at the end of the recordings by adding 10 μM of the selective AMPA receptor blocker 2,3-Dioxo-6-nitro-1,2,3,4-tetrahydrobenzo[*f* ]quinoxaline-7-sulfonamide (NBQX) to the bath. Recordings were performed in cells from slices incubated (15 min) with either vehicle or NGB-2904 (1 μM). Recordings were performed using a Multiclamp 700B/Digidata 1550A system (Molecular Devices, Sunnyvale, CA, USA) and digitized at a 10,000 Hz sampling frequency. All the electrophysiological recordings were analyzed using the Clampfit 10.6 software (Molecular Devices). AMPA receptor-mediated EPSC amplitude was calculated as the difference between the peak response and the baseline. NMDA receptor-mediated EPSC amplitude was calculated as the amplitude 50 ms after the response onset (Ahmad et al., 2012).

#### 2.4.2 Field recordings

Extracellular field recordings were conducted on 400 μm transverse hippocampal slices as described previously (Gulisano et al., 2019). After sectioning using a manual tissue chopper, slices were placed in a recording chamber and perfused (1–2 mL/min) with ACSF solution containing (in mM) 124 NaCl, 4.4 KCl, 1 Na_2_HPO_4_, 25 NaHCO_3_, 2 CaCl_2_, 2 MgCl_2_, and 10 glucose, maintained at 29 °C and continuously bubbled with 95% O_2_ and 5% CO_2_. After 120 min of recovery, field excitatory post-synaptic potentials (fEPSPs) were recorded in CA1 stratum radiatum using a glass electrode filled with ACSF in response to Schaffer collateral stimulation by a bipolar tungsten electrode. Basal synaptic transmission (BST) was assessed by stimulating with a series of increasing voltage pulses (from 5 to 35 V); fEPSP slope and afferent volley amplitude were measured (Torrisi et al., 2026). Before LTP induction, baseline responses were recorded every minute using a voltage that evoked a response of 35% of the maximum evoked response in BST. A theta-burst stimulation (TBS) protocol was used to induce LTP, consisting of trains of 10 bursts at 100 Hz with five pulses per burst and a 200 ms interburst interval at the test pulse intensity. Three TBS trains were delivered with a 15-s intertrain interval. LTP was recorded for 120 min after tetanus and fEPSP slopes were normalized to the first 15 min of baseline recordings. Recordings were performed using a Digidata 1440A system and analyzed using Clampfit 10 software.

### 2.5 Quantitative PCR (qPCR)

Total RNA was extracted from mouse hippocampal tissue using the RNeasy Mini Kit (Qiagen, Cat. No. 74104) according to the manufacturer's instructions. RNA concentration and purity were initially assessed using a NanoDrop spectrophotometer, and accurate quantification was subsequently performed using the Qubit RNA High Sensitivity Assay Kit (Thermo Fisher Scientific, Cat. No. Q32852). RNA samples were then normalized to the concentration of the sample with the lowest yield prior to reverse transcription. Complementary DNA (cDNA) was synthesized from normalized RNA samples using the QuantiTect Reverse Transcription Kit (Qiagen, Cat. No. 205311) following the manufacturer's protocol. Gene expression analysis was performed using specific primer pairs targeting Drd3 gene (Fw: 5′-GGGGTGACTGTCCTGGTCTA-3′; Rv: 5′-AAGCCAGGTCTGATGCTGAT-3′; product length 110 bp; acc. Num. NM007877.2). GAPDH was used as the reference housekeeping gene (Fw: 5′-CAACTCACTCAAGATTGTCAGCAA-3′; Rv: 5′-GGCATGGACTGTGGTCATGA-3′; product length 118 bp; acc. Num. NM001289726) (Leggio et al., 2021). Quantitative PCR was performed using the QuantiNova SYBR Green PCR Master Mix (Qiagen, Cat. No. 208052) on a LightCycler 480 system (Roche) using 384-well plates. Reactions were carried out in a final volume of 10 μL containing 1 μL of cDNA template and primers at a final concentration of 0.7 μM each. The PCR cycling conditions were as follows: initial activation at 95 °C for 2 min, followed by 40 cycles of denaturation at 95 °C for 5 s and combined annealing/extension at 60 °C for 10 s. A melting curve analysis was performed at the end of the amplification protocol to verify the specificity of PCR products. Gene expression levels were normalized to GAPDH.

### 2.6 Western blot

Western blot analyses were performed on hippocampal tissue obtained from the contralateral hemibrain of the same animals previously used for qPCR analyses and stored at −80 °C after tissue collection. Hippocampi from WT, 3xTg, and α7KO mice were manually homogenized in 200 μL of RIPA buffer (Thermo Fisher Scientific) supplemented with phosphatase and protease inhibitors (Thermo Fisher Scientific) at a 1:100 dilution and sonicated 3 times for 1 min, with a 1-min break between each cycle, on ice. Protein concentrations were determined using a bicinchoninic acid (BCA) assay. A total of 40 μg of protein per sample was loaded into 4–15% gradient SDS–PAGE gels (Bio-Rad). Proteins were then transferred into 0.22 μm nitrocellulose membranes (Amersham Biosciences, Buckinghamshire, UK). Membranes were blocked for 5 min, at room temperature (RT), using EveryBlot blocking buffer (Bio-Rad) before incubation with the following primary antibody: rabbit D3 Dopamine Receptor (extracellular) (Alomone Labs Cat# ADR-003, RRID:AB_2039830; 1:200). All membranes were incubated for 2 h at room temperature followed by overnight incubation at 4 °C. Mouse GAPDH (Abcam Cat# ab9485, RRID:AB_307275; 1:5000) was used as loading control. Membranes were incubated with the appropriate horseradish peroxidase (HRP)-conjugated secondary antibodies (Cell Signaling Technology; 1:2000) for 1 h at RT. Enhanced chemiluminescence (ECL) detection was performed using Westar Supernova (Cyanagen). Protein bands were visualized using the iBright imaging system (Thermo Fisher Scientific). Molecular weight estimation was carried out using Precision Plus Dual Color Protein Standards (Bio-Rad). Band intensities were quantified by densitometric analysis using ImageJ software (National Institutes of Health, Bethesda, MD, USA) and normalized to GAPDH immunoreactivity. Densitometric values obtained from technical replicates were averaged and the resulting mean value was used for statistical analyses and graphical representation.

### 2.7 Statistics

All experiments were performed in blind with respect to treatment or genotype. Data were expressed as mean ± standard error mean (SEM). The level of significance was set at *p* < 0.05.

After data collection, statistical analysis was performed by Systat 9 and GraphPad Prism Software 9.5.1. We used different tests, based on preliminary analyses of normal distribution by Shapiro-Wilk normality test: i) ANOVA for repeated measures to analyze BST and LTP; ii) one-way ANOVA with Bonferroni's *post-hoc* correction for residual potentiation of LTP, NOR, NOL and open field; iii) one sample *t*-test to compare D with zero in NOR and NOL; iv) two-way ANOVA for condition × sex to evaluate sex differences in NOR and NOL. AMPA/NMDA ratio was analyzed using one-way ANOVA followed by Tukey's *post hoc* test.

Given the non-normal distribution of qPCR and Western Blot measurements, as assessed by Shapiro-Wilk normality test, we used non-parametric Kruskal-Wallis test and Dunn's multiple comparison test.

## 3 Results

### 3.1 D3R blockade rescued different types of memory in AD animal models

Because hippocampal dysfunction is a major determinant of learning and memory deficits in AD, we investigated whether D3R-targeting compounds could improve cognitive performance in AD models. To this aim, we assessed recognition and spatial memory using the NOR and NOL tasks, two paradigms that critically rely on intact hippocampal and parahippocampal circuits and are highly sensitive to early AD-related dysfunction (Barker and Warburton, 2011; Leger et al., 2013).

The NOR task primarily evaluates the ability to discriminate a familiar from a novel object, reflecting recognition memory processes, whereas the NOL task tests spatial memory by assessing the detection of changes in object location.

**Figure 1:**
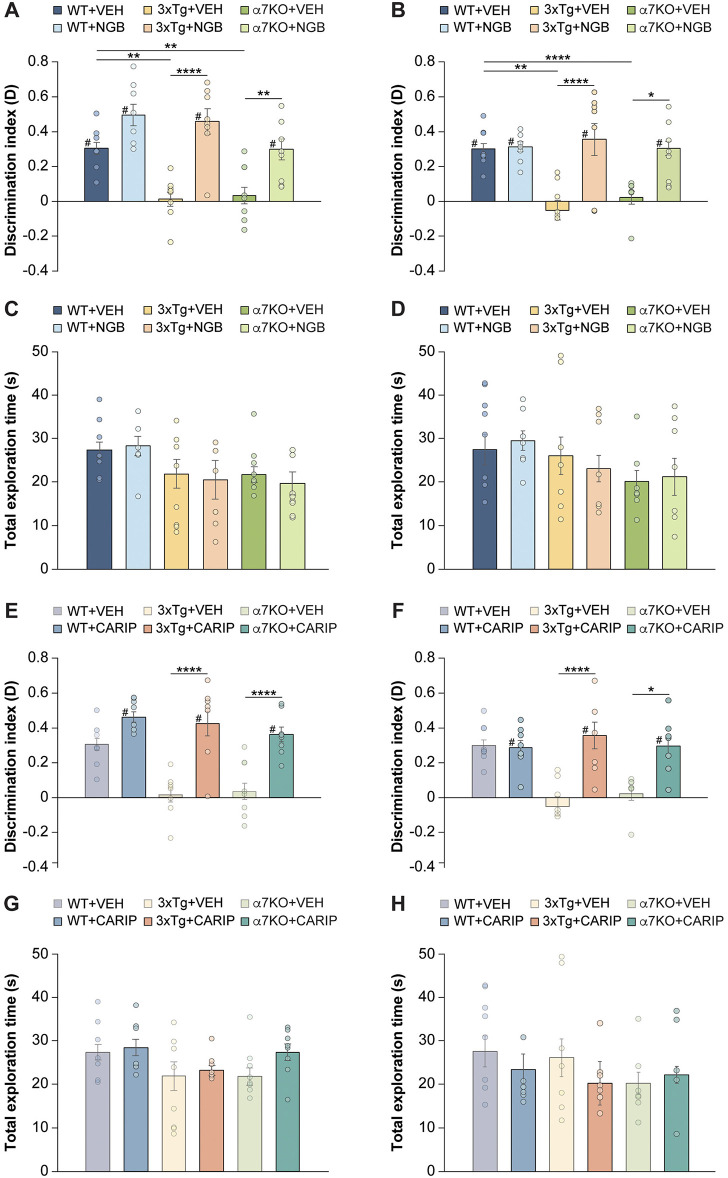
D3 receptor blockade rescues recognition and spatial memory deficits in AD mouse models. **(A)** Novel object recognition (NOR) task. Discrimination index (D) measured during the test phase (T2) in WT, 3xTg-AD, and α7KO mice treated with vehicle or NGB-2904 (acute i.p. 3 mg/kg). Both AD models showed impaired recognition memory compared with WT mice, which was rescued by NGB-2904 treatment. **(B)** Novel object location (NOL) task. Discrimination index measured during the test phase (T2) in WT, 3xTg-AD, and α7KO mice treated with vehicle or NGB-2904. NGB-2904 restored spatial memory performance in both AD models. **(C, D)** Total exploration time during the NOR **(C)** and NOL **(D)** tasks. No significant differences among experimental groups were observed, indicating that the behavioral effects were not due to changes in locomotor activity or exploratory drive. **(E)** NOR task following treatment with the D2/D3R antagonist cariprazine (acute i.p. 0.01mg/kg). Cariprazine improved recognition memory in both 3xTg-AD and α7KO mice. WT, 3xTg-AD and α7KO treated with vehicle are the same reported in **(A)**. **(F)** NOL task following treatment with cariprazine. Cariprazine restored spatial memory performance in AD mice. WT, 3xTg-AD and α7KO treated with vehicle are the same reported in **(B)**. **(G, H)** Total exploration time during the NOR **(G)** and NOL **(H)** tasks following cariprazine treatment, showing no differences among groups. WT, 3xTg-AD and α7KO treated with vehicle are the same reported in **(C)** and **(D)** for NOR and NOL, respectively. Data are presented as mean ± SEM. * *p* < 0.05; ** *p* ≤ 0.01; **** *p* ≤ 0.0001; # difference from 0. Statistical analyses are reported in the Results section.

Statistical analyses of the discrimination index [D = (exploration of novel object (or position)–exploration of familiar object (or position) / total exploration time] confirmed memory impairment in both AD-models (3xTg-AD and α7KO *n* = 10/10) compared with WT (*n* = 10) in NOR (one-way ANOVA with Bonferroni *post-hoc* test: 3xTg-AD vs. WT *p* = 0.002; α7KO vs. WT *p* = 0.005; Figure 1A) and NOL test (3xTg-AD, α7KO and WT = 8/8/8; 3xTg-AD vs. WT: *p* < 0.001; α7KO vs. WT: *p* = 0.015; Figure 1B). Indeed, AD mice spent a similar amount of time exploring the familiar and the novel object/position, and D was not different from zero (NOR: *p* = 0.746 for 3xTg-AD and *p* = 0.490 for α7KO; NOL: *p* = 0.379 for 3xTg-AD and *p* = 0.596 for α7KO), indicating a failure of learning, whereas D was significantly higher than zero in WT mice that showed normal long-term memory (*p* < 0.001 for both NOR and NOL).

To determine whether D3R blockade could rescue AD-related cognitive deficits, mice were treated with an acute i.p. injection of NGB-2904 20 min prior to T1 (NOR and NOL: 3xTg-AD *n* = 8/8, α7KO *n* = 9/10; WT *n* = 8/8). The treatment restored recognition and spatial memory in AD mice, which spent a greater amount of time exploring the novel object or the object in the novel position during T2 (analysis of D after treatment for NOR: 3xTg-AD *p* < 0.001; α7KO *p* = 0.009 vs. vehicle; Figure 1A; for NOL: 3xTg-AD <0.001; α7KO *p* = 0.023 vs. vehicle; Figure 1B). In both tasks, D became significantly different from zero after treatment (NOR: 3xTg-AD *p* < 0.001; α7KO *p* = 0.001; NOL: 3xTg-AD *p* = 0.004; α7KO *p* < 0.001). No alteration in performance was observed in WT mice following treatment with NGB-2904 (D vehicle vs. treatment NOR: *p* = 0.203; NOL: n = 8 *p* > 0.999; D different from zero: NOR: *p* < 0.001, NOL: *p* = 0.004; Figures 1A, B).

To further exclude potential confounding effects on exploratory activity, we analyzed the percentage of exploration directed toward each object during both the training (T1) and testing (T2) phases of the NOR and NOL tasks, as well as the total exploration time during T1. As expected, no significant preference for either object was observed during the training phase in any experimental group, confirming the absence of intrinsic object bias. During the testing phase, vehicle-treated 3xTg-AD and α7KO mice failed to discriminate between familiar and novel objects/locations, whereas NGB-2904- and cariprazine-treated animals displayed the expected preference for the novel object/location. Total exploration time during T1 did not differ among treatment groups, further supporting the conclusion that the behavioral effects observed in NOR and NOL performance were not secondary to alterations in exploratory drive. These data including statistics are reported in Supplementary Figure 2.

No differences among groups were detected in total exploration time in NOR and NOL (NOR: one-way ANOVA: *F*_(5, 49)_ = 1.750; *p* = 0.141; NOL: *F*_(5, 48)_ = 1.039; *p* = 0.406), indicating that the observed effects were not due to changes in locomotion or exploratory drive (Figures 1C, D).

To test whether cariprazine could reproduce the pro-cognitive effects observed with NGB-2904, a separate cohort of mice (3xTg-AD *n* = 9; α7KO *n* = 8) received an acute i.p. injection of the drug 30 min prior to T1. Cariprazine treatment improved both recognition and spatial memory in AD mice, as indicated by a significant increase in D compared with vehicle-treated AD animals (NOR: 3xTg-AD *p* < 0.001; α7KO *p* < 0.001 vs. vehicle; Figure 1E; NOL: 3xTg-AD *p* < 0.001; α7KO *p* = 0.047 vs. vehicle; Figure 1F). As observed with NGB-2904 treatment, D became significantly different from zero in cariprazine-treated mice (NOR: 3xTg-AD *p* < 0.001; α7KO *p* < 0.001; NOL: 3xTg-AD *p* = 0.002; α7KO *p* < 0.001).

In WT mice, cariprazine treatment did not significantly modify performance in either task (comparison with WT vehicle: NOR *n* = 9, *p* = 0.297; NOL *n* = 8 *p* > 0.999), and D remained significantly different from zero (NOR <0.001; NOL *p* < 0.001), consistent with intact memory in control animals (Figures 1E, F). Analysis of object exploration during both the training (T1) and testing (T2) phases confirmed the absence of object bias and showed that cariprazine did not alter exploratory behavior in WT mice (Supplementary Figure 2).

Similarly to NGB-2904, cariprazine did not affect total exploration time in either the NOR (*F*_(5, 50)_ = 2.252; *p* = 0.063) or NOL tasks (*F*_(5, 48)_ = 0.701; *p* = 0.626), indicating that the observed behavioral effects were not attributable to changes in locomotor activity or exploratory behavior (Figures 1G, H).

We also examined potential sex-dependent differences in behavioral performance within our experimental groups by stratifying the dataset according to sex. Although this analysis was limited by the relatively small number of animals, no significant sex-dependent differences were detected either in the impairment of memory in vehicle-treated 3xTg-AD and α7KO mice or in the behavioral positive effects induced by NGB-2904 or cariprazine (Supplementary Figure 3).

Overall, these results indicate that recognition and spatial memory deficits in both male and females AD mice are effectively reversed by treatment with D3R-targeting drugs, including the selective antagonist NGB-2904 and the D3-preferring compound cariprazine.

### 3.2 D3R blockade did not alter locomotor activity or anxiety-related behaviors

To determine whether the cognitive effects observed in the NOR and NOL tasks could be influenced by alterations in locomotor activity or anxiety-related behavior, mice were evaluated in the open field test.

**Figure 2:**
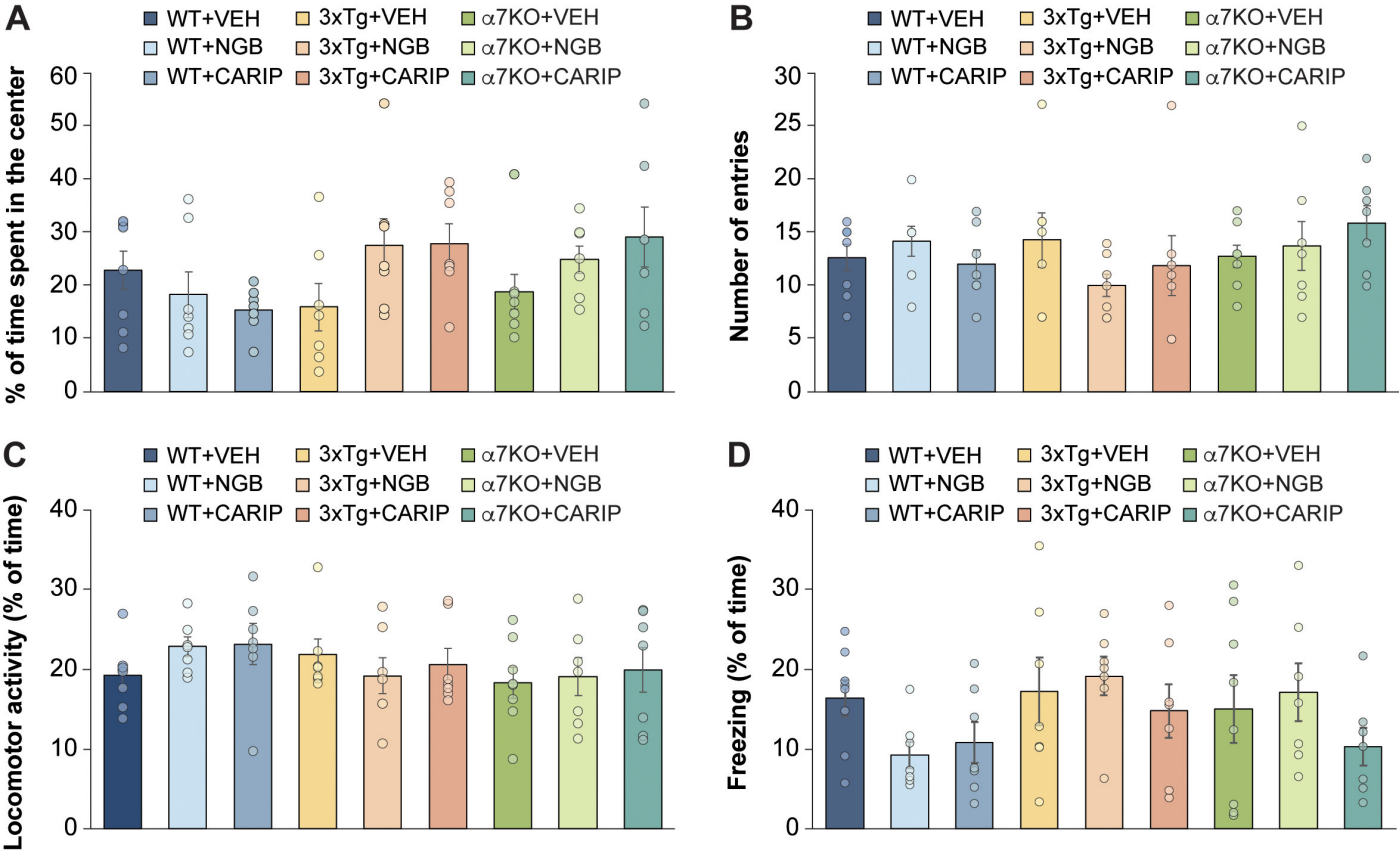
D3 receptor blockade does not affect locomotor activity or anxiety-related behavior in AD mouse models. **(A)** Time spent in the center of the open-field arena by WT, 3xTg-AD, and α7KO mice treated with vehicle, NGB-2904, or cariprazine. No significant differences among groups were detected. **(B)** Number of entries into the center of the arena in the same experimental groups. No significant differences among groups were detected, indicating comparable anxiety-related behavior. **(C)** Total exploration time in the open-field test. Neither genotype nor treatment significantly affected overall exploratory activity. **(D)** Freezing behavior measured during the open-field test. No significant differences were observed among groups. Data are presented as mean ± SEM. Statistical analyses are reported in the Results section.

No significant differences among experimental groups were detected in the time spent in the center of the arena (one-way ANOVA: *F*_(8, 56)_ = 1.763; *p* = 0.104; Figure 2A) or in the number of entries into the center (*F*_(8, 56)_ = 0.873; *p* = 0.545; Figure 2B), suggesting comparable anxiety-related behavior.

Similarly, total locomotor activity did not differ across groups (*F*_(8, 56)_ = 0.727; *p* = 0.667; Figure 2C), and no differences were observed in freezing behavior (*F*_(8, 56)_ = 1.234; *p* = 0.297; Figure 2D).

### 3.3 D3R blockade rescued the AMPA/NMDA ratio and basal synaptic transmission in AD models

To assess whether D3R blockade modulates excitatory synaptic strength in AD models, we first evaluated the AMPA/NMDA ratio using whole-cell patch-clamp recordings in hippocampal slices (Ripoli et al., 2014). This approach was based on our previous findings showing that pharmacological blockade of D3Rs with the selective antagonist NGB-2904 enhances AMPAR-mediated synaptic responses (Tropea et al., 2024).

**Figure 3:**
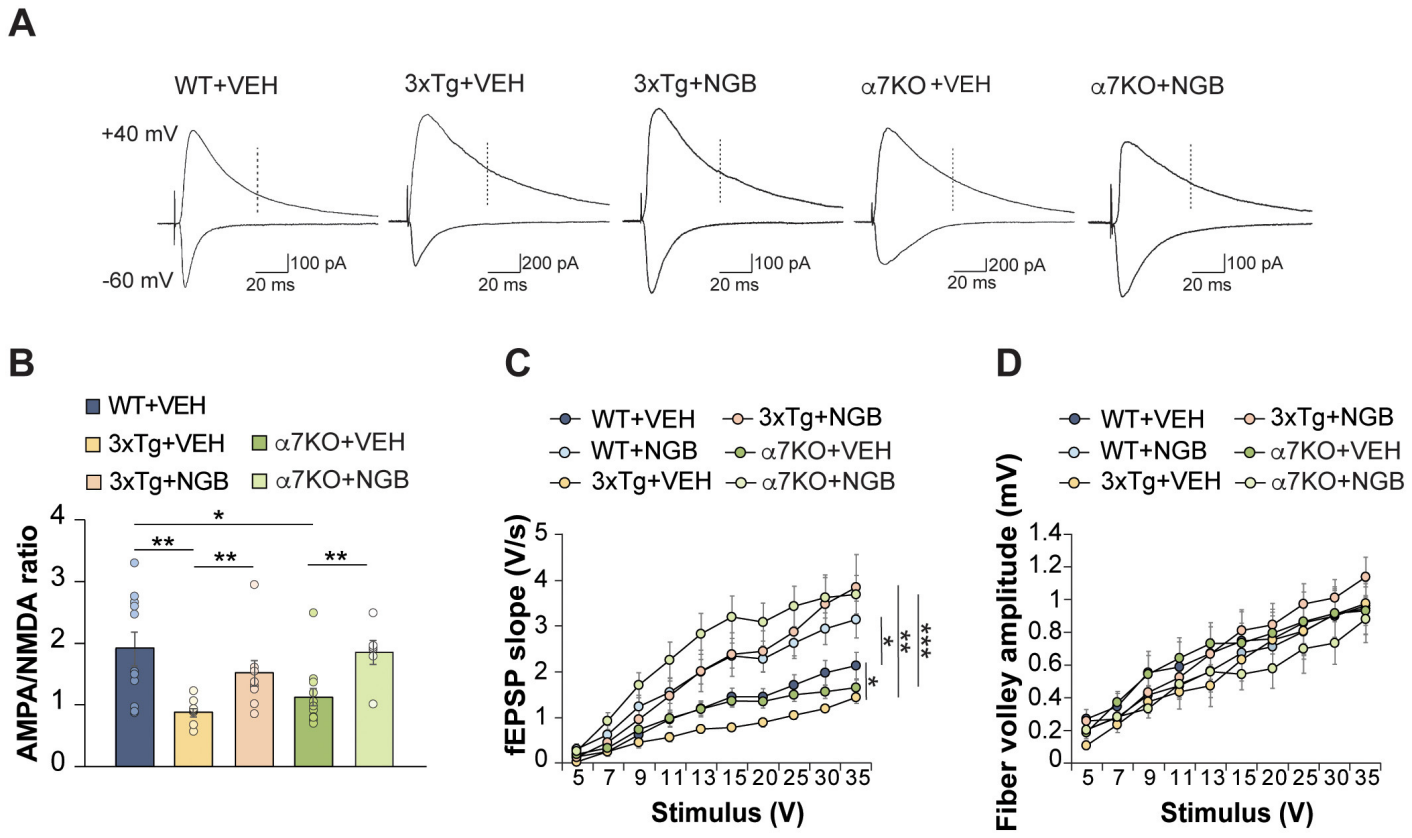
D3R blockade enhances excitatory synaptic transmission in hippocampal slices from AD mouse models. **(A)** Representative traces of AMPA receptor-mediated and NMDA receptor-mediated EPSCs recorded from CA1 neurons in hippocampal slices obtained from WT, 3xTg-AD, and α7KO mice after incubation with vehicle or the D3R antagonist NGB-2904 (1 μM). **(B)** Quantification of the AMPA/NMDA ratio under the experimental conditions shown in **(A)**. The ratio was calculated by measuring peak AMPAR-mediated EPSCs at −60 mV and peak NMDAR-mediated EPSCs at +40 mV. NGB-2904 restored the reduced AMPA/NMDA ratio observed in both 3xTg-AD and α7KO mice. **(C)** Basal synaptic transmission (BST) assessed by fEPSP input-output curves at CA3–CA1 synapses. Perfusion of hippocampal slices with NGB-2904 (1 μM) for 15 min prior to the I/O protocol increased fEPSP slope in WT, 3xTg-AD, and α7KO mice, rescuing the deficit observed in 3xTg-AD slices. **(D)** Fiber volley amplitude was not altered in either 3xTg-AD or α7KO mice and was not affected by NGB-2904 treatment. Data are presented as mean ± SEM. *p < 0.05, **p < 0.01, ***p < 0.001. Statistical analyses are reported in the Results section.

In the present study, the AMPA/NMDA ratio was significantly reduced in slices obtained from both 3xTg-AD (*n* = 9/4 cells/animals) and α7KO mice (*n* = 12/4 cells/animals) compared with age-matched WT controls (*n* = 11/4 cells/animals; one-way ANOVA: *F*_(4, 43)_ = 5.9, *p* < 0.001; Tukey's *post hoc* test: WT vs. 3xTg-AD, *p* = 0.008; WT vs. α7KO, *p* = 0.003), indicating a weakening of glutamatergic synaptic transmission in these models (Figures 3A, B). Treatment with NGB-2904 significantly increased the AMPA/NMDA ratio in both AD models (Tukey's *post hoc* test: α7KO vs. α7KO + NGB α7KO mice *n* = 7/3 cells/animals, *p* = 0.021; 3xTg-AD vs. 3xTg-AD + NGB *n* = 9/4 cells/animals, *p* = 0.047), restoring values toward those observed in WT animals (Figures 3A, B).We next recorded basal synaptic transmission (BST), assessed by fEPSP input/output curves in hippocampal slices, to evaluate the functional integrity and efficacy of excitatory synaptic connections and detect alterations in synaptic strength independent of activity-dependent plasticity. We observed a reduction in fEPSP slopes in 3xTg-AD (*n* = 10/4 slices/animals) compared with WT slices (*n* = 12/4 slices/animals) in repeated-measures ANOVA (*F*_(1, 20)_ = 6.855; *p* = 0.016), whereas no differences were detected between α7KO *n* = 11/4 slices/animals) and WT slices (*F*_(1, 21)_ = 0.290; *p* = 0.596) (Figure 3C). Afferent volley amplitude remained unchanged across genotypes (WT vs. 3xTg-AD: *F*_(1, 20)_ = 0.287; *p* = 0.598; WT vs. α7KO: *F*_(1, 21)_ = 0.001; *p* = 0.974) (Figure 3D). Treatment with NGB-2904 significantly increased fEPSP slopes in all genotypes, fully rescuing the deficit observed in 3xTg-AD mice (*n* = *F*_(1, 18)_ = 11.103; *p* = 0.004 between 3xTg-AD treated with vehicle or NGB-2904 *n* = 10/4 slices/animals). NGB-2904 further potentiated synaptic responses in slices from WT (*F*_(1, 21)_ = 7.204; *p* = 0.014; *n* = 11/4 slices/animals) and α7KO (*F*_(1, 20)_ = 15.746; *p* = 0.001; *n* = 11/4 slices/animals) (Figure 3C). D3R antagonism did not modify afferent volley amplitude, suggesting that its effect does not involve alterations in presynaptic glutamate release (WT vehicle vs. WT NGB-2904: *F*_(1, 21)_ = 0.151; *p* = 0.702; 3xTg-AD vehicle vs. 3xTg-AD NGB-2904: *F*_(1, 18)_ = 0.976; *p* = 0.336; α7KO vehicle vs. α7KO NGB-2904: *F*_(1, 20)_ = 1.151; *p* = 0.296; Figure 3D).

These results suggest that D3R blockade counteracts the AD-related impairment of excitatory transmission in the hippocampus.

### 3.4 D3R blockade rescued synaptic plasticity in AD models

**Figure 4:**
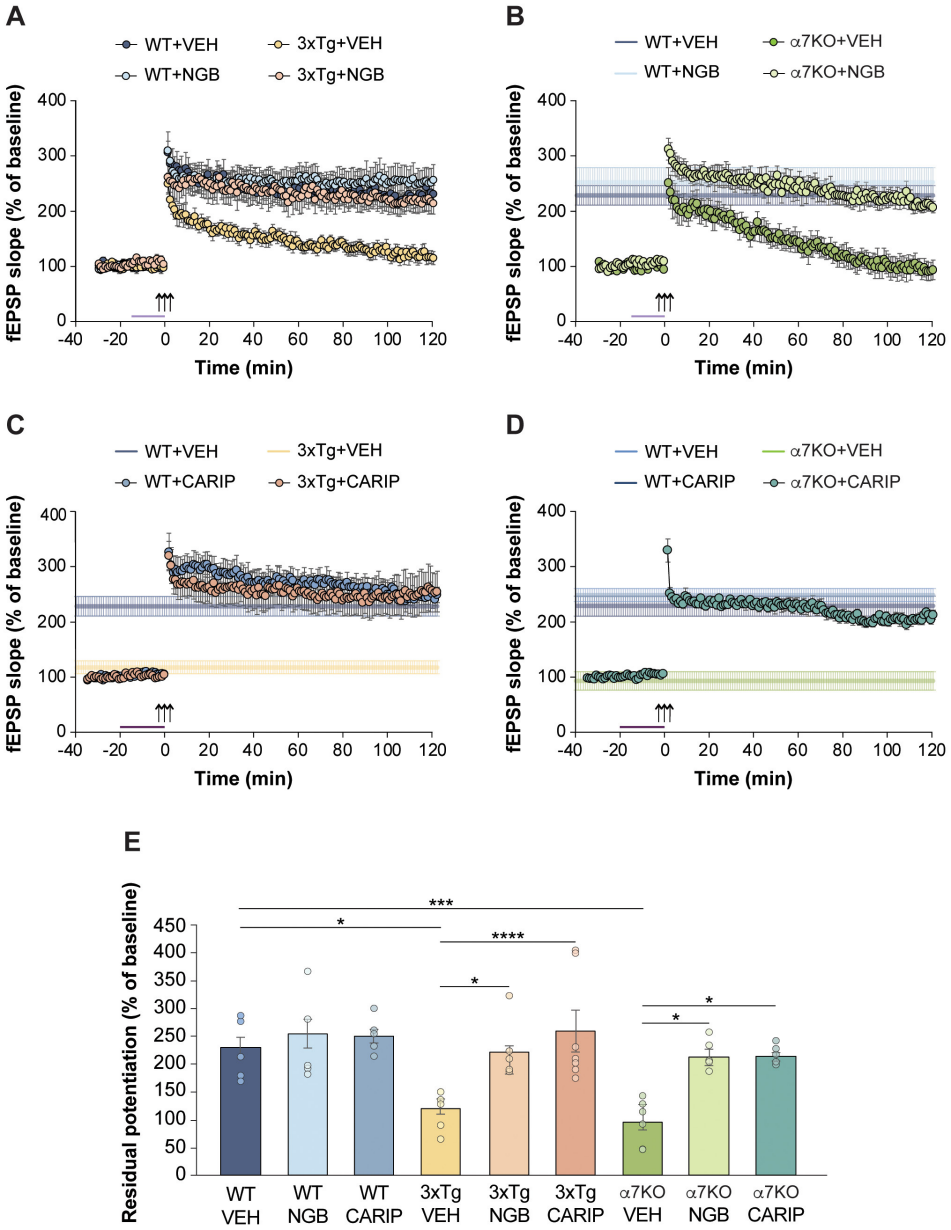
D3 receptor blockade restores long-term potentiation deficits in AD mouse models. **(A)** Hippocampal slices from WT and 3xTg-AD mice were perfused with vehicle or the D3 receptor antagonist NGB-2904 (1 μM) for 15 min prior to tetanic stimulation. LTP impairment observed in 3xTg-AD slices was restored by NGB-2904 treatment. **(B)** Similarly, NGB-2904 rescued LTP deficits in α7KO mice. Shaded area with dashed lines indicates the mean ± SEM of the last phase of LTP recording in WT + vehicle and WT + NGB-2904 shown in **(A)**. **(C)** Hippocampal slices from 3xTg-AD mice perfused with the D2/D3 antagonist cariprazine (1 nM) for 20 min prior to tetanic stimulation exhibited restored LTP. Shaded area with dashed lines indicates the mean ± SEM of the last phase of LTP recording in WT + vehicle and 3xTg-AD + vehicle shown in **(A)**. **(D)** Cariprazine rescued LTP impairment in slices from α7KO mice. Shaded area with dashed lines indicates the mean ± SEM of the last phase of LTP recording in WT + vehicle, and α7KO + vehicle shown in **(B)**, and WT + cariprazine shown in **(C). (E)** Quantification of residual potentiation (averaged over the last 5 min of LTP recording, 120 min after tetanus) confirmed the LTP impairment in 3xTg-AD and α7KO slices and its rescue following treatment with either NGB-2904 or cariprazine. Data are presented as mean ± SEM. *p < 0.05, ***p < 0.001, ****p < 0.0001. Statistical analyses are reported in the Results section.

Given the alterations in excitatory synaptic transmission observed in AD models and the well-established impairment of hippocampal LTP in both 3xTg-AD and α7KO mice (Oddo et al., 2003; Tropea et al., 2021), we next investigated whether acute pharmacological blockade of D3Rs could restore synaptic plasticity. We performed extracellular field recordings at CA3–CA1 synapses in hippocampal slices and first confirmed that LTP was impaired in both AD animal models, 3xTg-AD (repeated-measures ANOVA: *F*_(1, 12)_ = 17.444; *p* = 0.001 vs. WT; *n* = 7/5 slices/animals from WT; *n* = 7/4 slices/animals from 3xTg-AD) and α7KO mice (*F*_(1, 11)_ = 13.034; *p* = 0.004 vs. WT; *n* = 6/4 slices/animals from α7KO; Figures 4A, B, E). Hippocampal slices were then perfused with NGB-2904 for 15 min prior to LTP induction. Treatment with NGB-2904 rescued LTP in both models (*F*_(1, 11)_ = 12.212; *p* = 0.005, 3xTg-AD + vehicle vs. 3xTg-AD + NGB-2904, *n* = 6/4 slices/animals; *F*_(1, 10)_ = 18.500; *p* = 0.002, α7KO + vehicle vs. α7KO + NGB-2904, *n* = 6/4 slices/animals; Figures 4A, B, E). In WT mice, NGB-2904 treatment did not significantly affect LTP (*F*_(1, 12)_ = 0.196; *p* = 0.666, WT + vehicle vs. WT + NGB-2904, *n* = 7/5 slices/animals; Figures 4A, E).

To further confirm the beneficial effects of D3R blockade and to extend our findings to a clinically relevant compound, we next tested cariprazine, a D2/D3 receptor antagonist used in clinical practice as an antipsychotic, to determine whether it could ameliorate LTP deficits in AD animal models. Notably, cariprazine displays high affinity for D3Rs, and the dose used in our experiments was selected to preferentially target D3Rs (Kiss et al., 2010; Gyertyán et al., 2011). Treatment of hippocampal slices with cariprazine (20 min before tetanus) significantly improved LTP in 3xTg-AD (*F*_(1, 12)_ = 11.453; *p* = 0.005; *n* = 7/5 slices/animals from 3xTg-AD + cariprazine; Figures 4C, E) and α7KO models (*F*_(1, 10)_ = 21.721; *p* = 0.001; *n* = 6/4 slices/animals from α7KO + cariprazine; Figures 4D, E). Similarly to NGB-2904, cariprazine did not modify LTP in slices from WT mice (*F*_(1, 11)_ = 1.250; *p* = 0.287; *n* = 6/4 slices/animals from WT + cariprazine; Figures 4C, E). Analysis of residual potentiation (averaged over the last 5 min of LTP recordings, 120 min post-tetanus) confirmed that D3R antagonism rescued LTP impairment in AD mice (one-way ANOVA: *F*_(8, 49)_ = 7.996, *p* < 0.001; Bonferroni *post-hoc* correction: WT vs. 3xTg-AD *p* = 0.011; WT vs. α7KO *p* = 0.001; WT vs. 3xTg-AD + NGB 2904 *p* > 0.999; WT vs. 3xTg-AD + cariprazine *p* > 0.999; WT vs. α7KO + NGB 2904 *p* > 0.999; α7KO + cariprazine *p* > 0.999) (Figure 4E).

Together, these results demonstrate that both the selective D3R antagonist NGB-2904 and the D3-preferring compound cariprazine effectively restore impaired hippocampal synaptic plasticity in two distinct AD models.

### 3.5 Reduced D3R expression and PKA-dependent rescue of synaptic plasticity in 3xTg-AD and α7KO models

**Figure 5:**
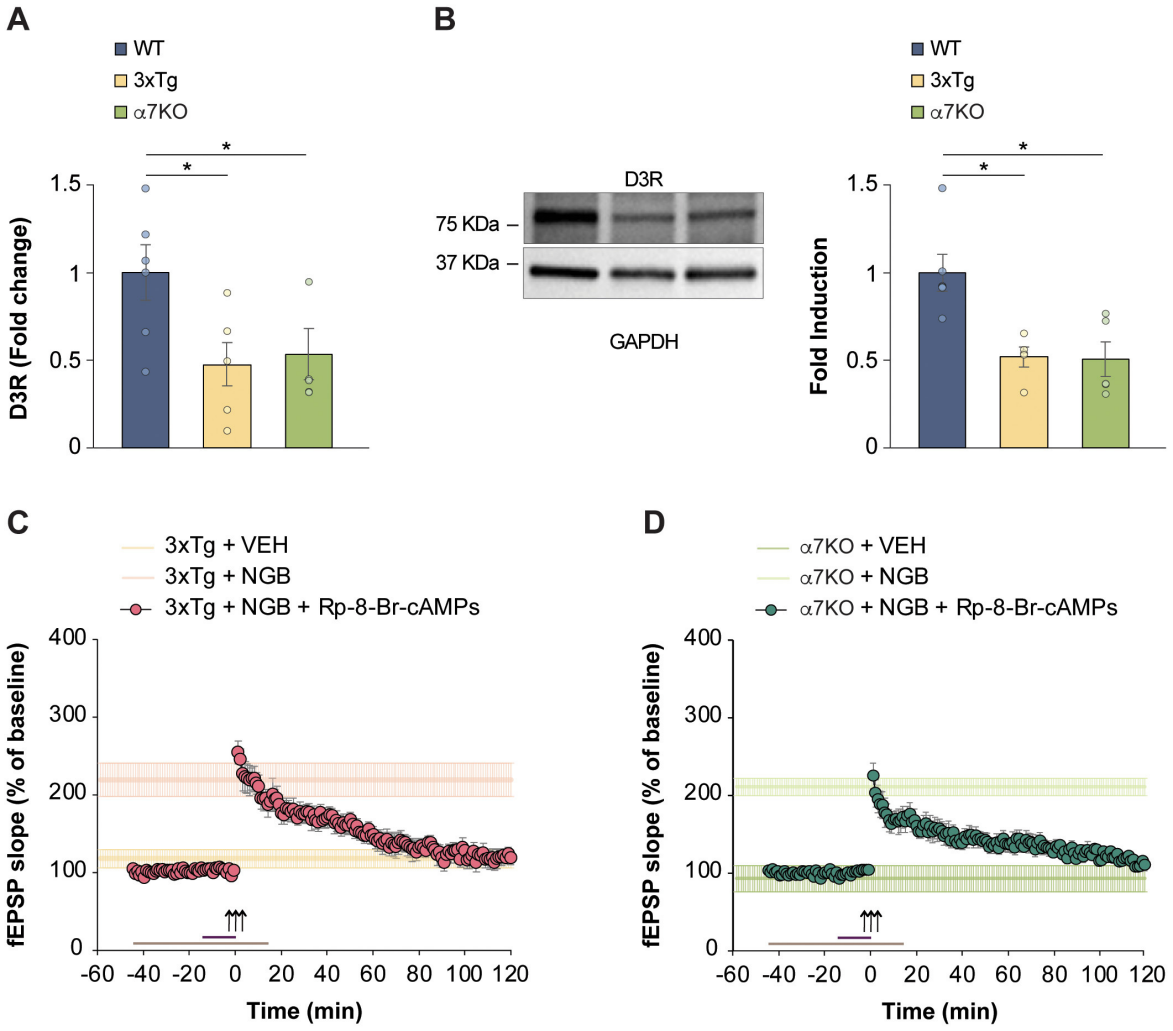
Altered hippocampal D3R expression and PKA-dependent rescue of synaptic plasticity. **(A)** Hippocampal D3R mRNA expression measured by qPCR revealed reduced transcript levels in both 3xTg-AD and α7KO mice compared with WT controls. Mean fold changes are expressed relative to WT levels. **(B)** WB analysis confirmed a reduction in D3R protein levels in both 3xTg-AD and α7KO hippocampi compared with WT (see Supplementary Figure 4 for original uncropped membranes). **(C)** Rp-8Br-cAMPs (20 μM, 30 min before and 15 min after tetanus) prevented the NGB-2904-induced rescue of LTP in both 3xTg-AD and **(D)** α7KO. Data are presented as mean ± SEM. *p < 0.05. Statistical analyses are reported in the Results section.

We first investigated whether the functional alterations observed in AD models were associated with changes in D3R mRNA levels or protein expression. The analysis of Drd3 mRNA levels in the hippocampus revealed a significant reduction of D3R transcripts in both 3xTg-AD (*n* = 5/5 hippocampi/animals) and α7KO mice (*n* = 5/5 hippocampi/animals) compared with WT controls (*n* = 6/6 hippocampi/animals; Kruskal-Wallis test with Dunn's *post-hoc* test: 3xTg-AD *p* = 0.029; α7KO *p* = 0.024; Figure 5A).

To determine whether these transcriptional changes were associated with altered receptor protein expression, we quantified hippocampal D3R protein levels by Western blot analysis. Consistent with the qPCR results, D3R protein expression was significantly reduced in both 3xTg-AD (*n* = 5/5 hippocampi/animals) and α7KO mice (*n* = 5/5 hippocampi/animals) compared with WT controls (*n* = 6/6 hippocampi/animals; Kruskal-Wallis test with Dunn's *post-hoc* comparisons: 3xTg-AD *p* = 0.0219; α7KO *p* = 0.0219; Figure 5B).

To investigate whether the facilitatory effects of D3R antagonism on synaptic plasticity were mediated by the cAMP/PKA signaling pathway, we examined the effect of the PKA inhibitor Rp-8-Br-cAMPS (20 μM) on NGB-2904-induced LTP rescue. Consistent with our previous findings in young adult WT mice, inhibition of PKA completely abolished the facilitatory effect of NGB-2904 in both AD models. In 3xTg-AD mice, co-application of Rp-8-Br-cAMPS prevented the NGB-2904-induced enhancement of LTP (*n* = 6/4 slices/animals; repeated-measures ANOVA: *F*_(1, 10)_ = 9.525, *p* = 0.012; Figure 5C). Similarly, PKA inhibition abolished the rescue of LTP produced by NGB-2904 in α7KO mice (*n* = 6/4 slices/animals; *F*_(1, 10)_ = 18.500, *p* = 0.002; Figure 5D).

These findings indicate that the beneficial effects of D3R antagonism require an intact cAMP/PKA signaling pathway and identify PKA as a critical downstream mediator of D3R-dependent modulation of hippocampal synaptic plasticity in both AD models.

## 4 Discussion

In this study, we demonstrated that pharmacological antagonism of D3Rs rescued hippocampal recognition and spatial memory, as well as synaptic transmission and plasticity, in two distinct mouse models of AD. Specifically, D3R antagonists improved memory performance in hippocampus-dependent behavioral tasks, normalized the AMPA/NMDA ratio and restored LTP. Notably, these behavioral and functional deficits occurred despite reduced D3R mRNA expression in both AD models, suggesting that altered receptor signaling rather than receptor overexpression contributes to synaptic dysfunction.

By comparing outcomes across 3xTg-AD and α7KO mice, we assessed whether modulation of D3R signaling mediates convergent benefits on hippocampal memory and synaptic plasticity across mechanistically distinct forms of AD.

A central finding of the present study is that pharmacological blockade of D3Rs restores hippocampal synaptic transmission and plasticity in AD models. The AMPA/NMDA ratio is widely considered a functional index of synaptic strength and postsynaptic AMPAR availability, as it reflects the relative contribution of AMPAR-mediated currents to excitatory transmission at glutamatergic synapses (Di Maio et al., 2016). Because activity-dependent insertion and stabilization of AMPARs at the postsynaptic membrane represent key mechanisms underlying synaptic potentiation, alterations in this ratio are closely linked to the capacity of synapses to express long-term plasticity. In the context of AD, converging evidence indicates that early synaptic dysfunction is driven by defects in AMPAR trafficking and retention, leading to reduced synaptic strength, impaired LTP, and cognitive decline (Penzes and Vanleeuwen, 2011; Henley and Wilkinson, 2016). Accordingly, the reduced AMPA/NMDA ratio and impaired LTP observed in both 3xTg-AD and α7KO mice are consistent with an early weakening of postsynaptic excitatory transmission, a hallmark of AD that precedes neurodegeneration. Restoration of AMPAR function, either by preventing receptor endocytosis or by promoting postsynaptic AMPAR stabilization, has been shown to ameliorate synaptic and cognitive deficits in experimental models of AD (Hsieh et al., 2006; Baglietto-Vargas et al., 2018; Eyvani et al., 2025). In line with this evidence, pharmacological antagonism of D3Rs with NGB-2904 increased the AMPA/NMDA ratio in hippocampal synapses from AD models, primarily through enhancement of AMPAR-mediated currents. This effect is consistent with our previous findings showing that D3R blockade strengthens excitatory synaptic transmission via postsynaptic mechanisms, including enhanced AMPAR function and increased GluA1 phosphorylation (Tropea et al., 2024).

Consistently, NGB-2904 also enhanced basal synaptic transmission, as reflected by the increase in fEPSP slope observed across genotypes and by the rescue of the deficit detected in 3xTg-AD mice. The reduction in fEPSP slope observed in 3xTg-AD slices occurred in the absence of changes in afferent volley amplitude, indicating preserved presynaptic fiber recruitment and suggesting that the deficit primarily reflects impaired postsynaptic responsiveness. This interpretation is further supported by the reduced AMPA/NMDA ratio observed in both AD models, pointing to a weakening of postsynaptic glutamatergic signaling. The ability of D3R blockade to enhance fEPSP responses is also consistent with our previous observations in aged mice, where pharmacological or genetic inhibition of D3Rs restored basal synaptic transmission through postsynaptic mechanisms (Tropea et al., 2024).

Together, these findings support the view that D3Rs exert a tonic inhibitory control over postsynaptic excitatory transmission in hippocampal circuits, a mechanism that may become maladaptive under pathological conditions. Pharmacological antagonism of D3Rs therefore appears to restore a synaptic state permissive for efficient glutamatergic transmission and LTP, ultimately supporting hippocampus-dependent memory processes.

Indeed, we showed here that pharmacological targeting of D3Rs restored both LTP and memory deficits in 3xTg-AD and α7KO mice. These findings extend our previous work demonstrating that pharmacological blockade or genetic deletion of D3Rs rescues synaptic plasticity and memory deficits associated with aging, also restoring the expression of plasticity related proteins through the potentiation of cAMP-PKA pathway (Tropea et al., 2024).

In contrast to the robust rescue of both basal synaptic transmission and LTP observed in AD models, NGB-2904 exerted a more selective effect in WT mice. While it significantly enhanced basal synaptic transmission, as indicated by the increase in fEPSP slope in the absence of changes in afferent volley amplitude, it failed to further potentiate LTP. These findings suggest that modulation of basal excitatory transmission and enhancement of activity-dependent synaptic plasticity are not necessarily coupled processes. While D3R antagonism can strengthen ongoing glutamatergic transmission, additional potentiation of LTP may require the recruitment of downstream signaling mechanisms that become progressively less responsive with aging or are already maximally engaged under conditions of strong synaptic stimulation. This interpretation is supported by previous evidence showing that dopaminergic modulation of hippocampal plasticity undergoes age-dependent alterations before impairments in synaptic function become detectable. Twarkowski et al. showed that the contribution of dopaminergic signaling to hippocampal LTP is altered in middle-aged animals even though the magnitude of LTP remains largely preserved. These findings suggest that age-related changes in neuromodulatory control may precede deficits in synaptic plasticity (Twarkowski and Manahan-Vaughan, 2016). Furthermore, previous studies have shown that increasing intracellular cAMP levels does not necessarily result in further enhancement of hippocampal LTP when potentiation is already robust. For example, phosphodiesterase inhibitors efficiently rescue impaired plasticity but produce little or no additional potentiation following strong tetanic stimulation protocols that already induce near-maximal levels of LTP (Gong et al., 2004; Vecsey et al., 2009). Together, these observations suggest that the capacity of dopaminergic/cAMP-dependent signaling pathways to further amplify synaptic potentiation may decline before the impairment in hippocampal plasticity become apparent.

Importantly, the rescue of LTP and memory deficits was observed not only following treatment with the selective D3R antagonist NGB-2904, but also after administration of cariprazine, a clinically approved antipsychotic displaying high affinity and preferential activity at dopamine D3Rs. The dose of cariprazine used in this study was selected to preferentially target D3Rs, consistent with the higher affinity of cariprazine for D3R relative to D2 receptors reported in previous pharmacological studies (Kiss et al., 2010; Gyertyán et al., 2011). Although cariprazine displays high affinity for D3Rs (low Ki), affinity alone does not determine the functional outcome of receptor activation. As a partial agonist with lower intrinsic efficacy than dopamine, cariprazine can act as a functional antagonist by competing with endogenous dopamine for receptor binding while producing a weaker receptor activation, thereby reducing overall D3R signaling. This mechanism has been proposed to contribute to the beneficial effects of cariprazine on cognitive function observed in several neuropsychiatric conditions (Calabrese et al., 2020; García-Fernández et al., 2024). Consistent with this interpretation, cariprazine has been reported to improve cognitive deficits in experimental models characterized by cholinergic or glutamatergic dysfunction (Neill et al., 2016; Zlatanova et al., 2022). In the present study, its ability to reproduce the synaptic and behavioral effects observed with the selective D3R antagonist NGB-2904 further supports a central role of D3R signaling in the rescue of synaptic plasticity and memory deficits. Nevertheless, cariprazine displays a broader pharmacological profile, including partial agonism at 5-HT1A receptors and lower-affinity interactions with 5-HT2 receptor subtypes, which have been implicated in cognitive processes (Briones-Aranda et al., 2025). While a contribution of these serotonergic targets cannot be entirely excluded, the low dose used in the present study, together with the convergence of effects observed with NGB-2904, supports a predominant role of D3R modulation in mediating the observed synaptic and behavioral effects.

In line with our findings, previous studies have shown that cariprazine improves performance across multiple cognitive domains in rodents (Barnes et al., 2018). Chronic administration of cariprazine has also been reported to induce adaptive changes in dopaminergic, serotonergic, and glutamatergic receptor systems across several brain regions (Caccia et al., 2013). In the present study, cariprazine was administered systemically, and therefore the precise neural substrates underlying its cognitive effects cannot be definitively identified. Nevertheless, the behavioral paradigms employed here critically depend on the coordinated activity of hippocampal, amygdalar, and prefrontal circuits, suggesting that the observed cognitive improvement may reflect integrated modulation of these networks.

From a preclinical perspective, the ability of pharmacologically distinct D3R-targeting compounds to consistently rescue synaptic plasticity and memory deficits across different AD models supports the notion that D3Rs represent a key modulatory node in hippocampal function. These findings highlight the relevance of dopaminergic mechanisms in early synaptic dysfunction and provide a strong experimental framework for further investigation of D3R signaling in neurodegenerative conditions. In this context, the efficacy of cariprazine is of particular interest, as it demonstrates that compounds with preferential activity at D3Rs can modulate synaptic and cognitive processes. However, the known risks associated with atypical antipsychotic treatments in elderly patients with dementia (Mok et al., 2024) suggest that these findings should be interpreted with caution. Rather than supporting immediate clinical translation, our results provide a preclinical rationale for further investigating D3R modulation as a strategy to counteract AD-related synaptic dysfunction and cognitive impairment.

These findings are consistent with previous studies showing that pharmacological antagonism of D3Rs improves several forms of learning and memory in rodents, including social memory, associative learning, and recognition memory (Loiseau and Millan, 2009; Millan et al., 2007, 2008; Sigala et al., 1997; Watson et al., 2012). Similarly, genetic deletion of D3Rs has been reported to enhance cognitive performance (Glickstein et al., 2005; Laszy et al., 2005; Xing et al., 2010). Although several studies have suggested that these pro-cognitive effects may involve cortical circuits (Loiseau and Millan, 2009), the specific contribution of D3Rs to hippocampal synaptic plasticity and memory processes has remained relatively unexplored. Our findings provide electrophysiological and behavioral evidence supporting a critical role for D3R signaling in regulating hippocampal synaptic function in the context of AD-related cognitive impairment.

Notably, D3R blockade did not modify total exploration time in the NOR and NOL tests, nor anxiety-related behavior in the open-field task. This indicates that the observed behavioral improvements were not secondary to changes in locomotor activity, motivation, or anxiety-like behavior, but rather reflect an enhancement of cognitive performance. This aspect is particularly relevant considering that D3Rs are known to regulate motivational, emotional, and locomotor processes in several brain circuits (Sokoloff and Le Foll, 2017).

Interestingly, both AD models displayed reduced hippocampal D3R expression at both the mRNA and protein levels despite showing robust electrophysiological and behavioral responses to pharmacological D3R blockade. This observation suggests that reduced receptor abundance does not preclude functional modulation of hippocampal circuits by D3R antagonists. The reduction observed at the transcript level was mirrored by a corresponding decrease in D3R protein expression, indicating that D3R downregulation in AD models extends beyond transcriptional changes and results in reduced receptor availability within the hippocampus. This finding is consistent with our previous observations showing an overall decrease in D3-positive profiles in the CA1 region during aging (Tropea et al., 2024), and with earlier reports describing a significant age-related decline in D3R mRNA expression in CA1 pyramidal neurons of human post-mortem brain tissue (Hemby et al., 2003). Notably, alterations of D2/D3R signaling have also been documented in AD patients, including reduced hippocampal D2/D3R availability detected by PET imaging (Kemppainen et al., 2003) and decreased D3R expression in post-mortem AD brains (Kumar and Patel, 2007).

Our previous ultrastructural analyses demonstrated that age-related D3R loss predominantly affected presynaptic glutamatergic elements, whereas postsynaptic D3Rs were largely preserved (Tropea et al., 2024). Importantly, the facilitatory effects of D3R antagonism are primarily mediated by postsynaptic mechanisms. Indeed, we showed that D3R blockade enhances AMPAR-mediated transmission and promotes structural strengthening of glutamatergic synapses, while leaving presynaptic glutamate release largely unaffected (Tropea et al., 2024). Thus, the relative preservation of postsynaptic D3Rs may explain why D3R antagonism remains effective despite the overall reduction in receptor expression, suggesting that the remaining receptor population retains sufficient functional capacity to support synaptic and cognitive responses.

In addition, preserved responsiveness despite reduced receptor expression may also be explained by the presence of receptor reserve, whereby substantial functional responses can be maintained even when only a fraction of the receptor population remains available (Kenakin and Christopoulos, 2013). Similarly, constitutive or basal receptor activity may allow the remaining D3Rs to exert a functionally relevant influence on hippocampal signaling despite their reduced abundance. Although these possibilities were not directly investigated in the present study, they may further contribute to explaining the preserved responsiveness to D3R antagonism despite reduced receptor expression.

To further investigate the mechanisms underlying this preserved responsiveness, we examined the contribution of the cAMP/PKA pathway and observed that pharmacological inhibition of PKA completely abolished the facilitatory effects of NGB-2904 on synaptic plasticity in both AD models. Together with our previous findings in young adult WT mice (Tropea et al., 2024), these results identify the cAMP/PKA cascade as a critical downstream mediator of D3R-dependent modulation of hippocampal plasticity. These findings might be considered in the broader context of the dopaminergic and cAMP-dependent deficits that characterize AD pathology. Several studies have demonstrated progressive degeneration of VTA dopaminergic neurons and reduced dopaminergic innervation of hippocampal and cortical regions in AD models (Nobili et al., 2017; Spoleti et al., 2024). In parallel, impairment of the cAMP/PKA/CREB signaling pathway is widely recognized as a central mechanism underlying synaptic dysfunction and memory impairment in AD. Aβ oligomers disrupt cAMP-dependent signaling, reduce PKA activity, impair CREB phosphorylation, and ultimately compromise the molecular processes required for LTP and memory formation (Vitolo et al., 2002). Consistent with these observations, reduced activity of multiple components of the cAMP signaling cascade has been reported in both AD patients and transgenic AD models, supporting the view that defective cAMP/PKA signaling represents a common molecular denominator of AD-related cognitive decline (for a review see Sanders and Rajagopal, 2020).

Since dopaminergic neurotransmission is an important upstream regulator of hippocampal cAMP/PKA-dependent plasticity, degeneration of dopaminergic inputs and impairment of cAMP/PKA signaling are likely to act synergistically in promoting synaptic dysfunction and memory decline in AD. Within this context, our findings suggest that D3R antagonism may exert its beneficial effects by enhancing the activity of a signaling pathway that remains pharmacologically accessible despite ongoing neurodegenerative changes. An additional possibility, albeit speculative, is that D3R antagonism may partially counteract the reduction in hippocampal dopaminergic tone reported in AD through modulation of presynaptic D3 autoreceptors. Future studies directly assessing dopamine release will be required to evaluate the contribution of this mechanism.

**Figure 6:**
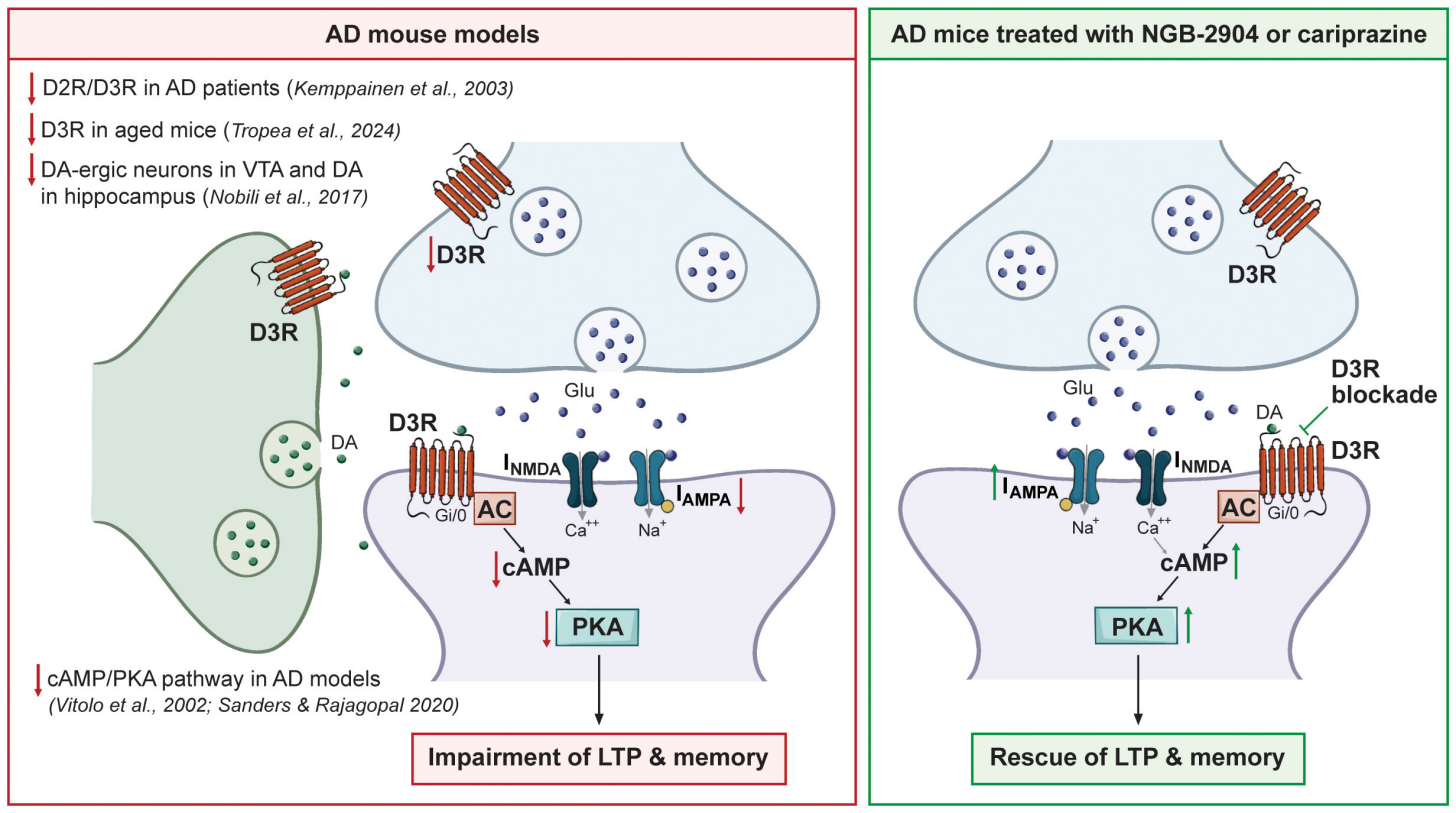
Proposed mechanism underlying D3R antagonist-mediated rescue in Alzheimer's disease models. Schematic representation of the working hypothesis emerging from the present study and supported by previous literature. In AD models, reduced dopaminergic tone and impaired cAMP/PKA signaling are accompanied by deficits in glutamatergic transmission, long-term potentiation (LTP), and memory. In parallel, both α7KO-AD and 3xTg-AD mice exhibit reduced hippocampal D3R expression at both the mRNA and protein levels. Despite this reduction, the residual D3R population remains functionally relevant, as demonstrated by the ability of D3R antagonism to restore synaptic plasticity and cognitive performance. Pharmacological blockade of D3Rs is proposed to enhance cAMP/PKA signaling, resulting in improved glutamatergic transmission and rescue of LTP and memory deficits. References indicated within the figure denote literature evidence supporting specific components of the proposed mechanism. This model is intended as a working hypothesis and does not imply direct experimental demonstration of all mechanistic steps.

Based on the present findings, we propose the working hypothesis illustrated in Figure 6, whereby D3R antagonism rescues synaptic plasticity and memory through restoration of cAMP/PKA-dependent signaling at glutamatergic hippocampal synapses in the context of AD-associated dopaminergic dysfunction.

In conclusion, our findings identify dopamine D3Rs as key modulators of hippocampal glutamatergic synaptic function and demonstrate that pharmacological blockade of these receptors rescues synaptic and cognitive deficits across two mechanistically distinct models of AD. These results support the emerging view that dopaminergic signaling contributes to early synaptic dysfunction in AD and highlight D3Rs as promising targets for strategies aimed at restoring hippocampal plasticity and cognitive function.

## Data Availability

The raw data supporting the conclusions of this article will be made available by the authors, without undue reservation.
