# Supplementary material for: Dopamine D3 receptor blockade restores hippocampal synaptic plasticity and rescues memory deficits in Alzheimer's disease mouse models
Source: Front Aging Neurosci. 2026 Jul 13;18:1840697. doi: 10.3389/fnagi.2026.1840697 (PMC13402449; doi:10.3389/fnagi.2026.1840697)
Supplement: Supplementary file 1 [file Data_Sheet_1.pdf]

Supplementary Material

1 Supplementary Figures

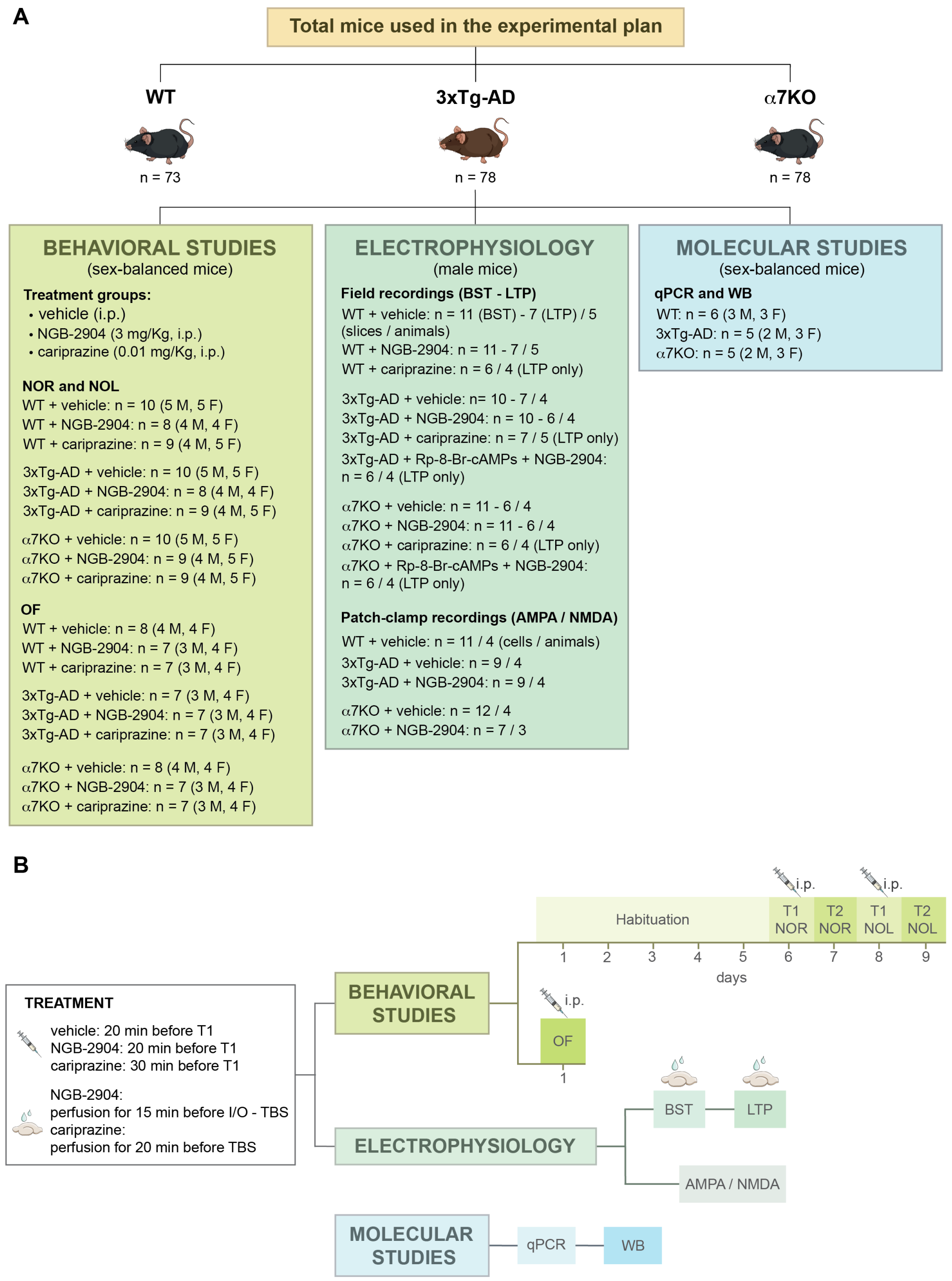

Supplementary Figure 1. Experimental design, animal allocation, and treatment schedule. (A) Summary of animal allocation across behavioral, electrophysiological, and molecular studies. The total number of WT, 3xTg-AD, and α7KO mice included in the study is indicated together with the number of animals used in each experimental paradigm. Behavioral experiments were performed in sex-balanced cohorts and included Novel Object Recognition (NOR), Novel Object Location (NOL), and

Open Field (OF) tests. Electrophysiological studies were conducted in male mice and included basal synaptic transmission (BST), long-term potentiation (LTP), and AMPA/NMDA ratio recordings. Molecular analyses were performed in sex-balanced cohorts and included quantitative PCR (qPCR) and Western blot (WB) experiments. For molecular studies, the two hippocampi obtained from each brain were processed separately, with one hippocampus used for qPCR analysis and the contralateral hippocampus used for Western blot experiments. Animals from each genotype were randomly assigned to the different experimental paradigms and treatment groups according to availability and experimental requirements. **(B)** Schematic representation of treatment schedules and experimental timelines. For behavioral studies, vehicle, NGB-2904 (3 mg/kg, i.p.), or cariprazine (0.01 mg/kg, i.p.) were administered before training sessions as indicated. For electrophysiological recordings, NGB-2904 or cariprazine were applied by bath perfusion before basal synaptic transmission, LTP, or AMPA/NMDA recordings according to the protocols described in the Methods section. Molecular analyses included assessment of hippocampal D3R expression by qPCR and Western blot.

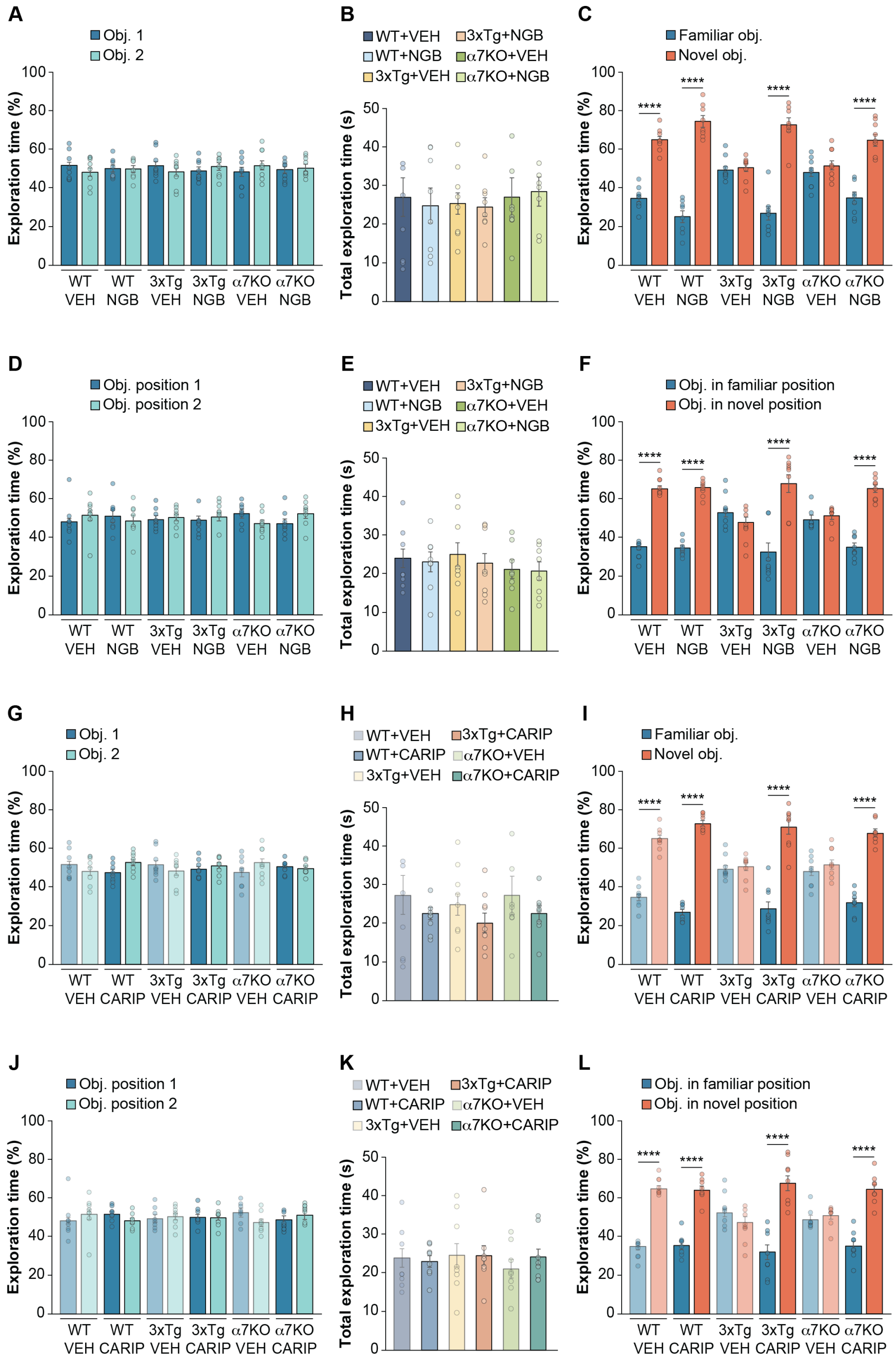

Supplementary Figure 2. Object exploration during training (T1) and testing (T2) phases of NOR and NOL tasks following D3R antagonist treatment. (A) Percentage of exploration time directed toward object 1 and object 2 during the training phase (T1) of Novel Object Recognition test in NGB-2904-treated mice and relative controls. During T1, both objects were identical and no significant preference for either object was detected in any group (WT + vehicle: n = 10, t = 1.185, p = 0.2514; WT + NGB-2904: n = 8, t = 0.09063, p = 0.9291; 3xTg-AD + vehicle: n = 10, t = 1.133, p = 0.2722; 3xTg-AD + NGB-2904: n = 8, t = 0.7528, p = 0.4641; α7KO + vehicle: n = 10, t = 1.020, p = 0.3214; α7KO + NGB-2904: n = 9, t = 0.3646, p = 0.7202). **(B)** Total exploration time during T1. One-way ANOVA revealed no significant differences among groups ($F_{(5,49)}$ = 0.1940, p = 0.9634). **(C)** Percentage of exploration time directed toward the familiar and novel objects during the testing phase

(T2). WT mice discriminated between familiar and novel objects (WT + vehicle: t = 12.29, $p < 0.0001$). In contrast, vehicle-treated 3xTg-AD and α7KO mice failed to discriminate between the two objects (3xTg-AD + vehicle: t = 0.4732, p = 0.6418; α7KO + vehicle: t = 1.020, p = 0.3214). NGB-2904 restored object discrimination in both 3xTg-AD and α7KO mice (3xTg-AD + NGB-2904: t = 9.064, $p < 0.0001$; α7KO + NGB-2904: t = 6.987, $p < 0.0001$). WT treated with NGB-2904 maintained a preferential exploration for the novel object (t = 11.47, $p < 0.0001$). **(D)** Percentage of exploration time directed toward position 1 and position 2 during T1 in Novel Object Location test in NGB-2904-treated mice and relative controls. During training, identical objects were placed in two equivalent locations and no significant preference for either position was detected in any group (WT + vehicle: n = 10, t = 0.8848, p = 0.3879; WT + NGB-2904: n = 8, t = 0.5314, p = 0.6035; 3xTg-AD + vehicle: n = 10, t = 0.3965, p = 0.6964; 3xTg-AD + NGB-2904: n = 10, t = 0.5839, p = 0.5665; α7KO + vehicle: n = 8, t = 1.944, p = 0.0723; α7KO + NGB-2904: n = 8, t = 1.540, p = 0.1459). **(E)** Total exploration time during T1. One-way ANOVA revealed no significant differences among groups ($F_{(5,48)} = 0.3529$, p = 0.8779). **(F)** Percentage of exploration time directed toward the familiar and novel locations during T2. WT mice discriminated between familiar and novel locations (WT + vehicle: t = 13.04, $p < 0.0001$). Vehicle-treated 3xTg-AD and α7KO mice failed to discriminate between the two objects (3xTg-AD + vehicle: t = 1.282, p = 0.2161; α7KO + vehicle: t = 0.7857, p = 0.4451). NGB-2904 restored location discrimination in both 3xTg-AD and α7KO mice (3xTg-AD + NGB-2904: t = 5.461, $p < 0.0001$; α7KO + NGB-2904: t = 10.36, $p < 0.0001$). WT treated with NGB-2904 maintained a preferential exploration for the object in the novel location (t = 15.24, $p < 0.0001$). **(G)** Percentage of exploration time directed toward object 1 and object 2 during T1 in Novel Object Recognition test in cariprazine-treated mice and relative controls. During T1, both objects were identical and no significant preference for either object was detected in any group (WT + cariprazine: n = 9, t = 2.303, p = 0.035; 3xTg-AD + cariprazine: n = 9, t = 0.8591, p = 0.403; α7KO + cariprazine: n = 8, t = 0.5182, p = 0.6124). WT, 3xTg-AD and α7KO treated with vehicle are the same reported in panel A. **(H)** Total exploration time during T1. One-way ANOVA revealed no significant differences among groups ($F_{(5,50)} = 0.8362$, p = 0.5303). WT, 3xTg-AD and α7KO treated with vehicle are the same reported in panel B. **(I)** Percentage of exploration time directed toward the familiar and novel objects during T2. Cariprazine restored object discrimination in both 3xTg-AD and α7KO mice (3xTg-AD + cariprazine: t = 8.386, $p < 0.0001$; α7KO + cariprazine: t = 11.83, $p < 0.0001$). WT treated with cariprazine maintained a preferential exploration for the novel object (t = 21.95, $p < 0.0001$). WT, 3xTg-AD and α7KO treated with vehicle are the same reported in panel C. **(J–L)** Novel Object Location (NOL) task in vehicle- and cariprazine-treated mice. **(J)** Percentage of exploration time directed toward position 1 and position 2 during T1 in Novel Object Location test in cariprazine-treated mice and relative controls. During training, identical objects were placed in the two locations and no significant preference for either position was detected in any group (WT + cariprazine: n = 8, t = 1.713, p = 0.1087; 3xTg-AD + cariprazine: n = 9, t = 0.02565, p = 0.9799; α7KO + cariprazine: n = 9, t = 0.8091, p = 0.4303). WT, 3xTg-AD and α7KO treated with vehicle are the same reported in panel D. **(K)** Total exploration time during T1. One-way ANOVA revealed no significant differences among groups ($F_{(5,48)} = 0.2758$, p = 0.9242). WT, 3xTg-AD and α7KO treated with vehicle are the same reported in panel E. **(L)** Percentage of exploration time directed toward the familiar and novel locations during T2. WT mice discriminated between familiar and novel locations (WT + vehicle: t = 13.04, $p < 0.0001$). Cariprazine restored location discrimination in both 3xTg-AD and α7KO mice (3xTg-AD + cariprazine: t = 6.599, $p < 0.0001$; α7KO + cariprazine: t = 8.549, $p < 0.0001$). WT treated with cariprazine maintained a preferential exploration for the object in the novel location (t = 9.723, $p < 0.0001$). WT, 3xTg-AD and α7KO treated with vehicle are the same reported in panel F. Data are presented as mean ± SEM. **** $p \leq 0.0001$.

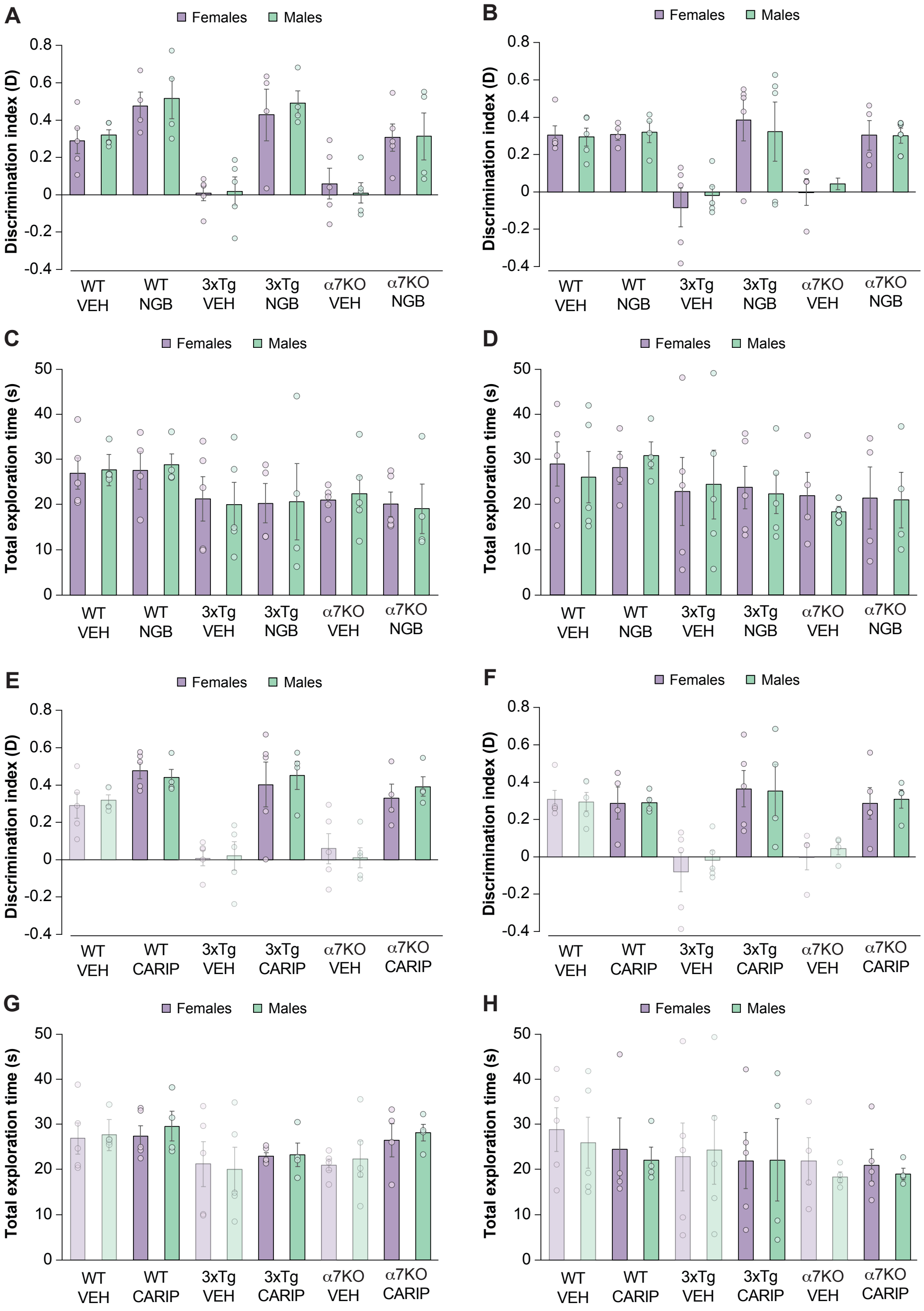

Supplementary Figure 3. The effects of D3R blockade on recognition and spatial memory are not influenced by sex in AD mouse models. (A) Novel object recognition (NOR) task following treatment with NGB-2904. Discrimination index (D) measured during the test phase (T2) in male and female WT, 3xTg-AD, and α7KO mice (WT + vehicle: n = 5/5 males/females; WT + NGB-2904: n = 4/4 males/females; 3xTg-AD + vehicle: n = 5/5 males/females; 3xTg-AD + NGB-2904: n = 5/5 males/females; α7KO + vehicle: n = 5/5 males/females; α7KO + NGB-2904: n = 4/5 males/females). Two-way ANOVA revealed no significant sex × condition interaction ($F_{(5,43)}$ = 0.121; p =0.987). **(B)** Novel object location (NOL) task following treatment with NGB-2904. Discrimination index (D) measured during the test phase (T2) in male and female WT, 3xTg-AD, and α7KO mice (WT + vehicle: n = 5/5 males/females; WT + NGB-2904: n = 4/4 males/females; 3xTg-AD + vehicle: n = 5/5

males/females; 3xTg-AD + NGB-2904: n = 5/5 males/females; α7KO + vehicle: n = 4/4 males/females; α7KO + NGB-2904: n = 4/4 males/females). Two-way ANOVA revealed no significant sex × condition interaction ($F_{(5,42)}$ = 0.155; p = 0.977). **(C)** Total exploration time during the NOR task following treatment with NGB-2904. Two-way ANOVA revealed no significant sex × condition interaction ($F_{(5,43)}$ = 0.0368; p > 0.999). **(D)** Total exploration time during the NOL task following treatment with NGB-2904. Two-way ANOVA revealed no significant sex × condition interaction ($F_{(5,42)}$ = 0.0960; p = 0.992). **(E)** NOR task following treatment with cariprazine. Discrimination index (D) measured during the test phase (T2) in male and female WT, 3xTg-AD, and α7KO mice (WT + vehicle: n = 5/5 males/females; WT + cariprazine: n = 4/5 males/females; 3xTg-AD + vehicle: n = 5/5 males/females; 3xTg-AD + cariprazine: n = 4/5 males/females; α7KO + vehicle: n = 5/5 males/females; α7KO + cariprazine: n = 4/4 males/females). Two-way ANOVA revealed no significant sex × condition interaction ($F_{(5,44)}$ = 0.175; p = 0.971). WT, 3xTg-AD and α7KO treated with vehicle are the same reported in panel A. **(F)** NOL task following treatment with cariprazine. Discrimination index (D) measured during the test phase (T2) in male and female WT, 3xTg-AD, and α7KO mice (WT + vehicle: n = 5/5 males/females; WT + cariprazine: n = 4/4 males/females; 3xTg-AD + vehicle: n = 5/5 males/females; 3xTg-AD + cariprazine: n = 4/5 males/females; α7KO + vehicle: n = 4/4 males/females; α7KO + cariprazine: n = 4/5 males/females). Two-way ANOVA revealed no significant sex × condition interaction ($F_{(5,42)}$ = 0.0856; p = 0.994). WT, 3xTg-AD and α7KO treated with vehicle are the same reported in panel B. **(G)** Total exploration time during the NOR task following treatment with cariprazine. Two-way ANOVA revealed no significant sex × condition interaction ($F_{(5,44)}$ = 0.071; p = 0.996). WT, 3xTg-AD and α7KO treated with vehicle are the same reported in panel C. **(H)** Total exploration time during the NOL task following treatment with cariprazine. WT, 3xTg-AD and α7KO treated with vehicle are the same reported in panel D. Two-way ANOVA revealed no significant sex × condition interaction ($F_{(5,42)}$ = 0.059; p = 0.998). Data are presented as mean ± SEM.

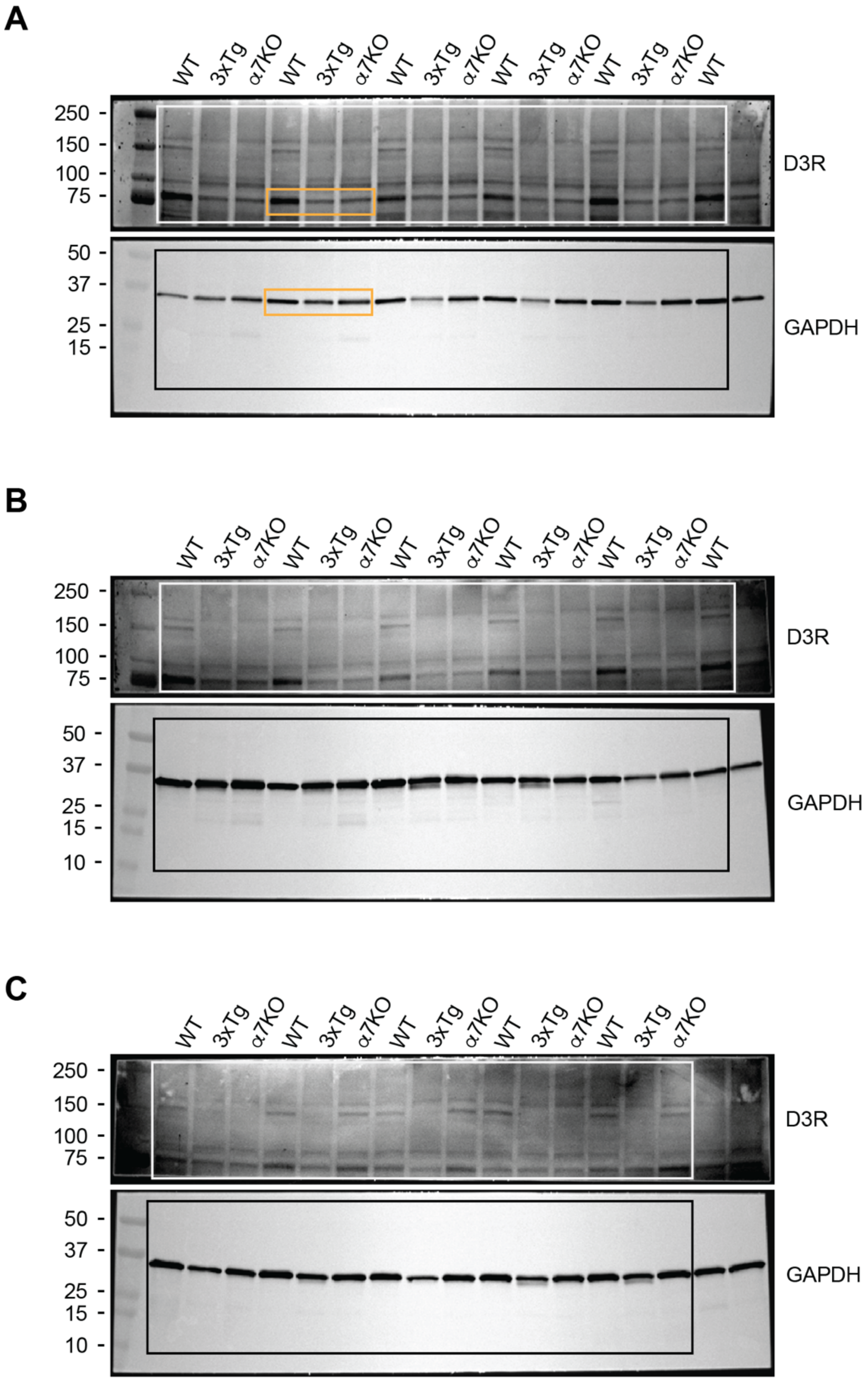

Supplementary Figure 4. Uncropped Western blots of hippocampal D3R expression in WT, 3xTg-AD, and α7KO mice. (A–C) Uncropped membrane images from three independent Western blot runs used for the quantification of D3R protein expression in hippocampal homogenates from WT,

3xTg-AD, and α7KO mice. D3R immunoreactivity and the corresponding GAPDH loading controls are shown. Western blot analyses were performed in technical replicates for each biological sample. White and black rectangles indicate the membrane regions used for densitometric quantification, whereas orange rectangles denote the representative bands displayed in Figure 5. Molecular weight markers (kDa) are shown on the left.
